# Assessing sediment toxicity risks with bioavailable metal fractions: new factors and index applied to the Colombian tropical Andes hotspot

**DOI:** 10.1007/s10653-025-02536-3

**Published:** 2025-05-22

**Authors:** Ingrid Vanessa Rincon-Vasquez, Nicola Fohrer, Daniel Rosado

**Affiliations:** 1 Department of Hydrology and Water Resources Management, Institute for Natural Resource Conservation, Kiel University, 24118 Kiel, Germany; 2 Department of Chemical and Environmental Engineering, Higher Technical School of Engineering, Universidad de Sevilla, Camino de los Descubrimientos s/n, 41092 Seville, Spain

**Keywords:** Trace elements contamination in hotspots, Heavy metal bioavailability, Sediment toxicity, Artisanal mining in Paramos, Sequential extraction, Environmental monitoring in Andes

## Abstract

Heavy metal toxicity risk assessments in sediments often rely on pseudototal concentrations, despite the higher theoretical predictive potential of bioavailable fractions. This study introduces the Bioavailable Fraction Toxicity Factor (BTf) and the Bioavailable Fraction Toxicity Index (BTI) to evaluate metal toxicity risks using a bioavailable fraction calculated as the sum of the first two steps of the Tessier sequential extraction procedure. Investigating heavy metal pollution (Cd, Cr, Cu, Mn, Ni, Pb, Zn) in the Vetas River catchment, a critical freshwater source in the Santurbán Páramo within the Tropical Andes biodiversity hotspot, the study identified artisanal and small-scale mining as the primary driver of contamination. Water and sediment of mining areas, particularly La Baja Creek and El Volcán Village, exhibited the highest concentrations of metals, with some sediment levels being categorized as strongly contaminated by the Geoaccumulation Index and Pollution Load Index and exceeding the Probable Effect Concentration threshold. Bioavailable fraction of metals in sediments were measured. Bioavailable fractions were higher in mining-affected areas, suggesting greater potential for metal release under acidic conditions. The BTf and BTI provided a more nuanced understanding of metal toxicity risks compared to pseudototal concentrations, with higher BTI values in mining-influenced sites. These findings underscore the need for mitigation measures to address heavy metal pollution and highlight the ecological importance of the Santurbán Páramo. Further research into bioremediation potential using local flora is recommended to support sustainable management practices.

**Supplementary Information:**

The online version contains supplementary material available at 10.1007/s10653-025-02536-3.

## Introduction

Heavy metal pollution in aquatic ecosystems poses a significant environmental challenge worldwide (Sojka & Jaskuła, 2022). These contaminants enter aquatic ecosystems through various natural and anthropogenic sources, including mining (Ali et al., 2019; Huang et al., 2020), tend to accumulate in sediments (Shou et al., 2022) and, depending on their bioavailability, they can bioaccumulate and biomagnify through the food chain. This process poses significant toxicity risks to both aquatic life and human health (Kachoueiyan et al., 2023, 2024a; Li et al., 2022). The assessment of heavy metal pollution in sediments often relies on indices that consolidate metal concentrations into a single numerical value, which facilitates communication (Kumar et al., 2019). These indices primarily focus on the accumulation of metals in sediments, highlighting enrichment relative to natural background levels, and their calculation typically involves two steps. First, the metal concentrations are normalized to a background or reference value, resulting in one value per metal per sample. Examples of this step include the geoaccumulation index (Müller, 1979) and the enrichment factor (Salomons & Förstner, 1984). Second, all the normalized values for a given sample are integrated into a single value, such as the pollution load index (Tomlinson et al., 1980).

In addition to reporting metal concentrations and enrichment levels, it is necessary to assess the potential toxicity risk to biota (Birch, 2017). To address this, researchers have established sediment quality guidelines that link specific metal concentrations with toxicity risks. These guidelines typically define low thresholds, below which adverse effects on organisms are rare, and high thresholds, above which toxic effects are likely (Birch, 2017; Karaouzas et al., 2021). Notable examples include the effect range low (ERL) and effect range median (ERM) developed by Long et al. (1995) and the threshold effect concentration (TEC) and probable effect concentration (PEC) developed by MacDonald et al. (2000). Some studies have utilized these guidelines as normalization values to propose new indices based on toxicity risk, using methodologies similar to those employed for assessing anthropogenic impacts on metal concentrations. Examples include the mean ERM quotient (mERMQ) and the mean PEL quotient (mPELQ) (Karaouzas et al., 2021; Long & MacDonald, 1998; Yavar Ashayeri & Keshavarzi, 2019).

Sediment quality guidelines traditionally rely on total metal content to assess toxicity risks. However, metals in sediments exist in different fractions, each with varying degrees of bioavailability. More bioavailable fractions are often more indicative of the potential toxicity effects of metals than less available ones (Fang et al., 2022; Gao et al., 2018; Sauvé et al., 2000; Valero et al., 2020). Currently, only a few indices consider the bioavailable fractions of metals in sediments to assess toxicity risks. Among these are the Risk Assessment Code (RAC) (Perin et al., 1985; Saeedi & Jamshidi-Zanjani, 2015) and the Modified Risk Assessment Code (mRAC) (Benson et al., 2018; Saeedi & Jamshidi-Zanjani, 2015).

This issue is particularly critical in areas impacted by artisanal mining, a significant source of heavy metals in aquatic ecosystems worldwide (Casso-Hartmann et al., 2022; Rivera-Parra et al., 2021). Such activities are particularly detrimental in biodiversity hotspots like those in Colombia (Dossou Etui et al., 2024), where informal, small-scale, and unregulated gold and silver mining generates large amounts of mining waste containing mercury (Hg) and sulfide minerals such as pyrite, chalcopyrite, and pyrrhotite. The oxidation of these sulfides can produce acid mine drainage (AMD) that is enriched with heavy metals (Hudson-Edwards & Dold, 2015; Wolff Carreño et al., 2005).

Frequently, artisanal mining takes place in the Colombian paramos, high-altitude moorlands located above the upper forest line and below the snowline (aprox 2800 and 4700 m) isolated in an archipelago-like distribution in the Andean mountains between latitudes of 11°N and 8°S covering approximately 35,000 km^2^ (Betancur-Corredor et al., 2018; Diazgranados et al., 2021; Llambí et al., 2019). Paramos have porous soils with a high-water retention capacity that stores and purifies surface and groundwater and serve as a freshwater source to major cities across the country (Buytaert et al., 2006; Cresso et al., 2020).

The Colombian paramos are part of the tropical Andes hotspot and can be considered a hotspot within this hotspot because of their high species endemism degree (Cuesta et al., 2017; Myers et al., 2000; Patiño et al., 2021). These ecosystems are home to approximately 4700 plant species (Torre et al., 2008) and 3431 vascular plants species (Madriñán et al., 2013). Paramos also serve as exceptional carbon sinks (Segura Madrigal et al., 2019). Along with other ecosystems, they contribute to making Colombia the most biodiverse country in the world relative to land area (Clerici et al., 2019; Gonzalez-Salazar et al., 2017).

The Vetas River catchment, located in the Eastern Cordillera of northeast Colombia, has experienced significant pressure due to a 400-year tradition of mining. The upper part of this area includes sections of the Santurbán páramo, a crucial hydrological and biological hotspot (Loaiza et al., 2020). Informal and small-scale gold and silver mining in the area (Corporación Geoambiental Terrae 2018) have produced elevated concentrations of As in water and sediments (Alonso Contreras, 2014; Alonso et al., 2020), as well as Mn and Cu in water (Jiménez-García & Palacio-Carreño, 2016; Wolff Carreño et al., 2005). These concentrations significantly exceeded the drinking water threshold established in Colombian regulations by the Resolution 2115 of 2007 (Ministry of Housing of Colombia, 2007), as well as those from the United States Environmental Protection Agency (USEPA, 2024). The Santurbán páramo is a critical ecological area where the Vetas, Charta, Tona, and Frio rivers originate. This region supplies approximately 30% of the freshwater to Bucaramanga, a major city in Colombia (Duarte-Abadía et al., 2023).

Despite the longstanding impact of mining, there is still a lack of comprehensive studies on the Vetas River that examine a wider variety of trace elements, hindering a thorough assessment of the mining impacts on the ecosystem and the implementation of effective protection measures (Loaiza et al., 2020).

This study introduces the bioavailable fraction toxicity factors and the bioavailable fraction toxicity index for heavy metals in sediments. These metrics are designed to integrate the risks associated with heavy metals in sediments into a single figure, using the threshold effect concentration (TEC) and the probable effect concentration (PEC) established by MacDonald et al. (2000). Furthermore, this study aims to determine the heavy metal pollution (Cd, Cr, Cu, Fe, Mn, Ni, Pb, and Zn) in sediment and water of the Vetas River catchment to evaluate their potential toxicity risks to biota. Given the artisanal and small-scale mining activities prevalent in the area, this study hypothesizes that the presence of mining activities is directly associated with elevated concentrations of heavy metals in both water and sediments. This area comprises a significant portion of the Santurbán Páramo, a region of high biodiversity and part of the Tropical Andes biodiversity hotspot.

In addition, this study reports on metal concentrations of bioavailable fractions (exchangeable and bound to carbonates) in sediments of the Vetas River catchment using the method proposed by Tessier et al. (1979) as described in Table S2. To the best of the authors' knowledge, this is the first time this method has been applied in this region in the scientific literature, providing crucial insight into the potential bioavailability and bioconcentration of trace elements in sediments. Ultimately, this study enhances communication about the risks associated with heavy metals in sediments for aquatic biota.

## Materials and methods

### Study area: Vetas River Basin

**Fig. 1:**
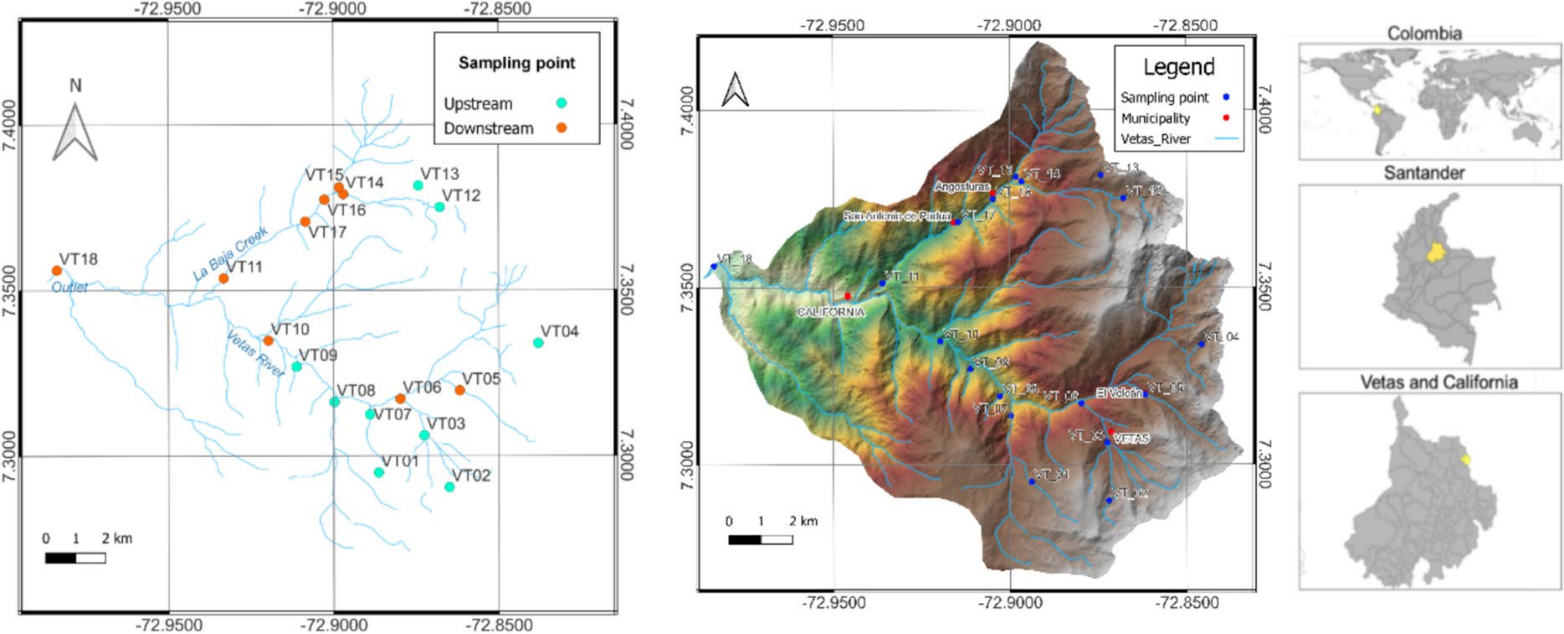
Location of study area and sampling points in Colombia (Vetas River: VT01-VT10, La Baja Creek: VT11-VT17, Outlet: VT18). Points are categorized into two groups: those upstream or in micro basins without mining activities in the Vetas River (VT01-VT04, VT07-VT09) and La Baja Creek (VT12, VT13) marked with blue dots, and those in mining districts in the Vetas River (VT05, VT06, VT10) and La Baja Creek (VT11, VT14-VT17) or downstream (outlet, VT18) marked with orange dots. VT05 is located in El Volcán Village. Coordinates in Table S1

The Vetas River basin is located approximately 50 km northeast of Bucaramanga in the Santander department (northeastern Colombia). The catchment is situated in two municipalities: Vetas and California (Fig. 1). The river is the main tributary of the Suratá river and flows from the Santurbán paramo at 4255 m above sea level (m) down to Suratá municipality at 1656 m. The water from its main tributaries, the Paez, La Baja and Mongora creeks, is utilized by local communities for mining, agriculture, and drinking (Alonso et al., 2020).

According to the Vivero Suratá weather station, maintained by the Institute of Hydrology, Meteorology and Environmental Studies of Colombia (IDEAM in spanish), the annual precipitation in the study area ranged from 660 to 1,200 mm between 1968 and 2021. The region experiences a bimodal rainfall pattern, with two rainy seasons occurring from March to mid-May and from mid-September to November, and two dry seasons occurring from December to March and from June to mid-September (IDEAM, 2024). The average daily maximum temperature is 35 °C in the lower areas, while the average daily minimum temperature in the páramo regions is 0 °C. The average relative humidity in the basin is 81%. The evaporation rate ranges from 700 to 1500 mm per year, while evapotranspiration varies between 910 and 1400 mm per year. Due to its tropical location, these values remain relatively stable throughout the year (IDEAM, 2024).

The Vetas River basin, including La Baja Creek, is well known for its extensive mining history, with numerous mines and processing plants frequently located along its banks, extending into the lower regions of the Santurbán Páramo (Cotula & Perrone, 2024; Flórez et al., 2022; Güiza-Suárez & Kaufmann, 2024; Morales Méndez & Rodríguez, 2016; Zárate Rueda et al., 2023; Zárate-Rueda et al., 2022). According to the 2011 census of mining units, California and Vetas had 147 and 78 mining units, respectively, being 34% in California and 82% in Vetas operating without a license (Ministry of Mines & Energy of Colombia, 2012). Although the Colombia 1930 Act of 2018 (known as the Páramos Law) prohibits mining, exploration, and exploitation in páramos (Congress of Colombia, 2018), the exact boundaries of the Santurbán páramo remain unclear (Duarte-Abadía & Boelens, 2016). The lack of regulation in exploitation and waste disposal practices of unlicensed mining poses a significant threat to the water quality of the Vetas River (Pardave Livia & Beltran Aguilar, 2007; Sojka & Jaskuła, 2022).

Moreover, between 64 and 96% of rocks in the Vetas catchment are classified as high potentially acid-generating (PAG) rock (MINESA, 2019), which is a high sulfidation epithermal deposit with pyrite as the primary ore mineral, followed by copper sulfides such as chalcopyrite, chalcocite, bornite, and covellite (MINESA, 2019). PAG rocks can generate sulfuric acid when exposed to weathering conditions, potentially dissolving metals and other harmful substances from the rocks (Hudson-Edwards & Dold, 2015). Additionally, MINESA (2019) estimated that mine tailings may contain approximatelly 50,8 g As, 2,7 g Bi 0,77 g Cd, 139 g Pb, 23,1 g Sb, 2,88 g Te, 2,1 g Th, 19 g U and 38 g Zn per ton. These concentrations can cause irreversible changes in the ecosystem and water quality.

### In situ parameters, sampling and sample pre-treatment

The sampling campaign was conducted in March 2022 and included 18 sampling points in water bodies (VT01-VT18). Ten in the Vetas River upstream of the confluence with La Baja Creek (VT01-VT10), seven in la Baja Creek (VT11-VT17) and one in the Vetas River downstream of the confluence with La Baja Creek (VT18), referred to as the outlet in this article.

To assess potential mining contamination in these areas, sampling points were divided into two groups. The first group included points located in areas unaffected by mining activity, either upstream or in micro basins without mining activity in both, the Vetas River (VT01-VT04, VT07-VT09) and La Baja Creek (VT12 and VT13). The second group included points located either in mining districts along the Vetas River (VT05, VT06, VT10) and La Baja Creek (VT11, VT14-VT17) or downstream (VT18). VT05 is located in El Volcán Village. VT02 and VT13 correspond to two lakes unaffected by mining, situated in the upper part of the basin in the Paramo de Santurbán, while remaining points correspond to creeks and rivers (Fig. 1).

No sediment samples were collected at points VT09 and VT13 due to strong currents and a lack of fine sediments. In addition, two tap water samples were collected from the municipal administrative centers of Vetas and California. In total, 20 water samples and 16 sediment samples were collected.

In situ water quality parameters (pH, conductivity, dissolved oxygen, and temperature) were measured at each sampling point at a depth of 10–20 cm. Measurements were taken using a WTW® Multi 3630 IDS portable multiparameter device, calibrated with standard solutions prior to use.

Samples were collected according to the guidelines outlined in the international standard ISO 5667 series (ISO 2020). Water samples were collected in 100 ml polyethylene bottles that had been previously washed and dried in the laboratory. At each sampling point, bottles were rinsed three times with the sample water before collection, filled completely (without air bubbles), and hermetically sealed. After collection, the water samples were stored at 4 °C in the dark (ISO 2020). Sediment samples from the top 5 cm were collected using a shovel across a cross-section of the river, avoiding areas with signs of leakage or surface disturbance. These samples were placed in hermetically sealed plastic bags and stored in a dark cooler at 4 °C. Sediment samples were dried at room temperature in an empty, clean, and closed room without air circulation due to the lack of an on-site oven. Once dried, the samples were sieved with a plastic sieve to a 2 mm size. Finally, the samples were transported to Germany for analysis at the laboratories of Kiel University.

### Heavy metal measurement in water samples

Water samples were filtered through 0.45 μm pore Teflon filters, acidified with a drop of Suprapur® nitric acid and stored at 4 °C until analysis. Metal concentrations in water were measured using a Thermo Scientific ® ICP-OES iCAP 6000 Series, which supports both axial and radial views. Calibration of the instrument was performed using Certipur® ICP multi-element standard solution IV and XIII from Merck, which were diluted at ratios of 1:50, 1:100, and 1:200.

For quality control and assurance (QC/QA), each measurement batch incorporated a procedure blank (reagents without sample), a duplicate sample (randomly selected for each batch) and a reference water (ERM-CA615) from the certified reference materials catalog of the European Commission's Joint Research Centre (JRC).

### Pseudototal content of heavy metal in sediment samples

Sediment samples were initially dried at 60 °C for 24 h, weighed, dried for an additional two hours until constant weight, disaggregated with an agate mortar, and finally sieved to obtain a fraction with a particle size < 100 μm.

Next, 0.2 g of each sediment sample was digested in 5 ml of Suprapur® nitric acid using a closed digestion block (Picotrace) at 140 °C for 16 h. The digestate was then filtered into 50 ml volumetric flasks and stored in 50 ml plastic bottles at 4 °C until analysis.

For QC/QA purposes, each digestion batch included a procedure blank (reagents without sample), a duplicate sample (randomly selected for each batch) and a reference soil (SO-4) from the Canadian Certified Reference Materials Project. Recovery rates for all elements consistently ranged between 95 and 105% and, therefore, were considered satisfactory (Kachoueiyan et al., 2024b).

Heavy metal concentrations in the digestates were measured with a Thermo Scientific ® ICP-OES iCAP 6000 Series, following the same procedure described for the water samples.

#### Geoaccumulation Index (Igeo)

The Geoaccumulation Index (Igeo) is a measure used to assess metal pollution in aquatic sediments and solid waste materials. Introduced by Müller (1979) and further detailed by Förstner and Müller (1981), evaluates the degree of contamination based on natural background concentrations (Förstner & Müller, 1981; Kowalska et al., 2018). The Igeo is expressed as the logarithmic transformation of the ratio between the metal concentration in a sample and a reference concentration, accounting for possible variations due to lithologic changes (Förstner & Müller, 1981). It is regarded as one of the most accurate contamination indices (Kowalska et al., 2018).

The formula for calculating Igeo is:

$$ {\text{Igeo}} = \log_{2} \frac{{X_{{{\text{Sample}}}} }}{{1.5 \times X_{{{\text{Background}}}} }} $$

where X_Sample_ is the concentration of an element in the sample. X_Background_ is the average concentration in the upper continental crust or the background value of the examined element in the area. 1.5 is a constant included to minimize errors due to lithologic variations.

The sampling point VT02 was adopted as the background value of this study for calculating both the geoaccumulation index and the pollution load index. This choice was made because no anthropogenic influence was detected at this site, providing a more accurate baseline for the study area than average reference values of element concentrations in the upper continental crust, such as those established by Turekian and Wedepohl (1961).

Igeo categorizes sediment quality into seven categories based on the degree of contamination of individual elements as follows (Förstner & Müller, 1981): category I (uncontaminated, ≤ 0), category II (uncontaminated to moderately contaminated, 0 < Igeo < 1), category III (moderately contaminated, 1 < Igeo < 2), category IV (moderately to strongly contaminated, 2 < Igeo < 3), category V (strongly contaminated, 3 < Igeo < 4), category VI (strongly to extremely contaminated, 4 < Igeo < 5) and category VII (extremely contaminated, Igeo ≥ 5).

#### Pollution load index (PLI)

The Pollution Load Index (PLI) was originally developed by Tomlinson et al. (1980) to address challenges in assessing heavy metal pollution in estuaries. It provides a measure of the contamination degree from a set of metals based on the Contamination Factor (CF), which is the ratio of the metal concentration in the sample to the background or reference concentration (Alharbi et al., 2023; Haynes & Zhou, 2022; Huang et al., 2020; Tomlinson et al., 1980).

The PLI is calculated as the n^th^-root of the product of the n-CF, where n represents the number of metals considered as shown below (Haynes & Zhou, 2022; Huang et al., 2020). In this study, n = 6.

$$\text{PLI }= {{(CF}_{Cd}*{CF}_{Cu}*{CF}_{Cr}* {CF}_{Ni}* {CF}_{Pb}* {CF}_{Zn})}^\frac{1}{6}$$

Similar to the Igeo calculation, the sampling point VT02 was used as the background value for calculating both the Igeo and PLI.

There is no consensus regarding the categorization of the PLI. According to Tomlinson et al. (1980), PLI > 1 indicates contamination. For a deeper interpretation of the results, this study adopts the following categorization (Aftab & Hakeem, 2022; Jorfi et al., 2017): No pollution (PLI ≤ 1), moderate pollution (1 < PLI ≤ 2), heavy pollution (2 < PLI ≤ 3), extremely high pollution (PLI > 3).

#### Comparison to guidelines

Reference concentrations of heavy metals were based on the Consensus-Based values developed by MacDonald et al. (2000): threshold effect concentration (TEC) and probable effect concentration (PEC) thresholds. The TEC represents the concentration below which adverse effects are not expected to occur, in mg/kg: Cd (0.99), Cr (43.4), Cu (31.6), Pb (35.8), Ni (22.7), Zn (121). The PEC indicates the concentration above which adverse effects are expected to occur more often than not, in mg/kg: Cd (4.98), Cr (111), Cu (149), Pb (128), Ni (48.6), Zn (459) (MacDonald et al., 2000).

#### Statistical analysis: principal component analysis (PCA) and correlation analysis

Principal Component Analysis (PCA) was performed to identify and understand the relationships among the measured variables. PCA is widely used to assess heavy metal pollution in soils because it reduces the dimensionality of the data and reveals the main sources of variation (Aidoo et al., 2021; Fagbenro et al., 2021; Huang et al., 2020; Iordache et al., 2022; Liu et al., 2013; Wei et al., 2011). The Pearson correlation coefficient was also calculated to define associations and dependencies between elements. This method is commonly used to evaluate heavy metal pollution in the environment (Fagbenro et al., 2021; Huang et al., 2020; Iordache et al., 2022). The Kaiser–Meyer–Olkin (KMO) test and Bartlett's test were conducted to evaluate the suitability of the data for principal component analysis (PCA) and correlation analysis prior to performing these analyses. A KMO value greater than 0.5 and a p-value from Bartlett’s test less than 0.05 are considered satisfactory, confirming the appropriateness of the data for the mentioned statistical analyses (Kachoueiyan et al., 2024b; Mishra et al., 2018). R studio software version 2023.06.1 + 524 (R Development Core Team, 2023) was used to produce the graphs and to perform statistical analysis.

### Sequential extraction for bioavailable metals in sediments, bioavailable fraction toxicity factor and bioavailable fraction toxicity index

The first and second steps of the sequential extraction method proposed by Tessier et al. (1979) were performed to obtain the exchangeable and carbonates fractions from sediments. Heavy metals were sequentially extracted from 1 g of sediment using solutions and conditions listed in Table S2. The metal content in the resulting extracts was measured with a Thermo Scientific ® ICP-OES iCAP 6000 Series instrument as described for water samples.

This work developed the bioavailable fraction toxicity factor (BTf) and the bioavailable fraction toxicity index (BTI). The BTf normalizes the sum of metal content in the exchangeable and carbonate-bound fractions according to the protocol defined by Tessier et al. (1979), which is equivalent to the acid-extractable fraction defined by the BCR-701 method (Rauret et al., 2001) as stated before (Casalino et al., 2013). This normalization uses sediment toxicity guidelines developed by MacDonald et al. (2000), specifically the threshold effect concentration (TEC) and the probable effect concentration (PEC). The BTf is calculated according to the following equations:

$$ {\text{If }}C_{m,s} < {\text{TEC}}_{m} \;{\text{then BTf}}_{m,s} = C_{m,s} /{\text{TEC}}_{m} \;{\text{and }}0 < {\text{BTf}}_{m,s} < 1 $$

$$ {\text{If TEC}}_{{\text{m}}} \le {\text{C}}_{{{\text{m}},{\text{s}}}} < {\text{PEC}}_{{{\text{m}} }} {\text{then BTf}}_{{{\text{m}},{\text{s}}}} = {1} + \left( {{\text{C}}_{{{\text{m}},{\text{s}}}} - {\text{TEC}}_{{\text{m}}} } \right)/\left( {{\text{PEC}}_{{\text{m}}} - {\text{TEC}}_{{\text{m}}} } \right){\text{ and 1}} \le {\text{BTf}}_{{{\text{m}},{\text{s}}}} < {2} $$

$$ {\text{If C}}_{{{\text{m}},{\text{s}}}} \ge {\text{PEC}}_{{\text{m}}} \;{\text{then BTf}}_{{{\text{m}},{\text{s}}}} = {2} + \left( {{\text{C}}_{{{\text{m}},{\text{s}}}} - {\text{PEC}}_{{\text{m}}} } \right)/{\text{PEC}}_{{\text{m}}} ){\text{ and BTf}}_{{{\text{m}},{\text{s}}}} \ge {2} $$

where C_m,s_ is the concentration of the metal *m* in the sample *s*, TEC_m_ is the TEC for the metal m, BTf_m,s_ is the bioavailable fraction toxicity factor of the metal *m* in the sample *s*, PEC_m_ is the PEC of the metal m.

The toxicity factors are interpreted as no sediment toxicity (Tf < 1), possible sediment toxicity (1 ≤ Tf < 2) and probable sediment toxicity (Tf ≥ 2) according to the interpretation of the TEC and PEC developed by MacDonald et al. (2000).

The bioavailable fraction toxicity factors of one sediment sample were integrated into the bioavailable fraction toxicity index (BTI) to express the toxicity of multiple metals in a single figure. The BTI is the geometric mean of the BTfs of one sample:

$$ {\text{BTI}}_{{\text{s}}} = \, \left( {{\text{BTf}}_{{{\text{m1}},{\text{s}}}} {\text{x BTf}}_{{{\text{m2}},{\text{s}}}} {\text{x }} \ldots {\text{ x BTf}}_{{{\text{mi}},{\text{s}}}} } \right)^{{{1}/{\text{i}}}} $$

where BTI_s_ is the toxicity index in the sample *s*, BTf_m1,s_ is the contamination factor of the metal *m1* in the sample *s*, and BTf_mi,s_ is the contamination factor of the i^th^ metal *mi* in the sample *s*. BTI levels are divided into three categories, similarly as it was done before for the BTf.

## Results and discussion

### In-situ parameters

The pH values in the study area varied substantially, with a range of approximately 5 units, while conductivity also showed significant variation, spanning about 1762 µS/cm (Table S3). Most pH measurements fell between 6.84 and 7.89, which are suitable for drinking water according to European Union (European Parliament, 2020) and Colombian (Ministry of Housing of Colombia, 2007) guidelines. These values also align with standards for the preservation of aquatic life set by the USEPA (2023) and comply with Colombian regulations for the discharge of mining waters into water bodies (Ministry of Environment & Sustainable Development of Colombia, 2015). However, three samples located in areas visibly affected by mining exhibited acidic pH values that could be considered outliers in El Volcán village (VT05, 4.48), and La Baja Creek (VT14, 2.78; VT17, 5.02). Their pH values are similar to those recorded in severely affected areas near tailings and spillage points in the Escalera, Chipchilla, Hoachocolpa, and Atoccomarca rivers in Peru, where Cacciuttolo and Cano (2022) reported pH values between 2.5 and 3.2. They are also comparable to those in the Odiel and Tinto rivers in Spain, where Olías et al. (2004) recorded pH values between 2.5 and 6.3. Point VT14 can be categorized as having extremely acidic mine water (Nordstrom et al., 2000).

The conductivity values had an average of 254.8 µS/cm and a standard deviation of 409.0 µS/cm, indicating a higher degree of variability compared to pH. Ten sampling points had conductivity values below 100 µS/cm, two were between 100 and 300 µS/cm, five ranged from 300 to 450 µS/cm, and one exceeded 1500 µS/cm. The three pH outliers also had higher-than-average conductivity values and were among the top five highest: VT05 (350 µS/cm), VT14 (1787 µS/cm), and VT17 (377 µS/cm). All these waters would be suitable for drinking according to the conductivity criteria of the European Union (European Parliament, 2020), but VT14 would not meet the Colombian guidelines (Ministry of Housing of Colombia, 2007).

Lower-than-average pH values and higher-than-average conductivity values were primarily associated with areas impacted by mining or located downstream (Figure S1).

Dissolved oxygen levels varied by only ~ 1 mg/L and consistently remained above 7.68 mg/L, indicating a high level close to saturation that can support aquatic life requiring oxygen. These values align with the typically high levels of oxygen observed in the upper sections of rivers, where steeper relief and the presence of more water jumps promote oxygenation (Ji et al., 2017). Additionally, lower population density in these areas reduces pollution from untreated sewage, which can deplete oxygen levels in water (Ji et al., 2017). Furthermore, mining activities have a minimal influence on oxygen concentrations (Karaca et al., 2018).

Temperature varied by approximately 9 °C, with this variation primarily attributed to changes in altitude, as indicated by a correlation coefficient of *r* =  − 0.7.

The two tap water samples collected from the municipal administrative centers of Vetas and California showed pH levels of 7.27 and 7.09, conductivity of 149 and 158 µS/cm, and dissolved oxygen levels of 7.47 and 7.52 mg/L, respectively. These measurements comply with all the aforementioned regulations, indicating that, based on these water quality parameters, the tap water is suitable for drinking.

### Heavy metal in water samples

Most of the metal concentrations in the samples were low, often below detection limits (Table 1). However, the drinking water threshold levels for all metals were exceeded in at least one sampling point. Mn, Ni and Fe were the most frequently above threshold levels, posing the greatest potential health risks for individuals consuming this water. Sampling points in mining areas of El Volcán village (VT05), La Baja Creek (VT14), the outlet (VT18) as well as, unexpectedly, an area in the upper part of the basin not traditionally associated with mining activities (VT01) exceeded the limits the most often. In particular, Fe and Ni exceeded limits at all four points, while Cu, Mn, and Pb exceeded limits at three points, and Cr and Zn at two points. At the outlet, Zn reached a significantly elevated concentration of 72.94 mg/L. Notably, elevated concentrations of Cu, Fe, Ni, Pb, and Zn were also found at point VT01.

**Table 1:**
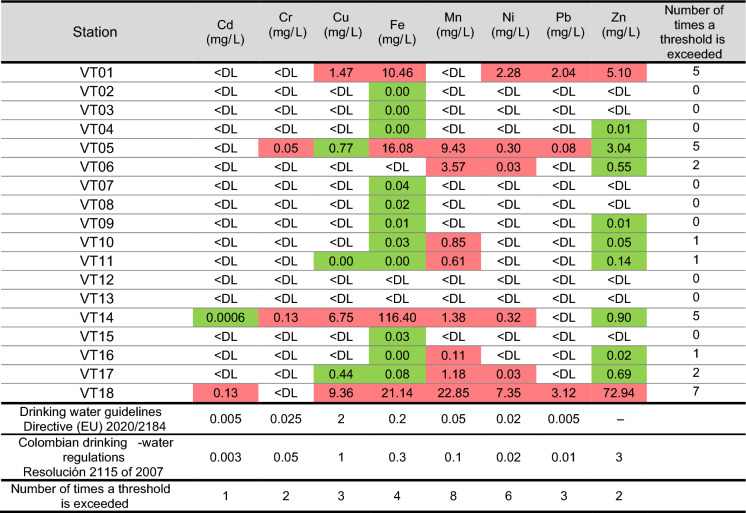
Concentration of Cd, Cr, Cu, Fe, Mn, Ni, Pb, and Zn in water samples of the Vetas River basin, Colombia, and guideline values for drinking water provided by European Union Directive 2020/2184 and the Colombian Resolution 2115 of 2007 (Ministry of Housing of Colombia, 2007)

Reddish cells indicate concentrations exceeding at least one of these guidelines. Green cells indicate non-exceed concentrations. < DL = below the detection limit

These values in mining districts and downstream were similar to those found in severely affected mining areas in the Odiel (Olías et al., 2004) river in Spain, as well as in Minera Caudalosa in Peru (Cacciuttolo & Cano, 2022) as shown in Table S4 in the supplementary material.

Water from the Vetas River and La Baja Creek is often used for irrigation and to supply households in rural areas through artisanal aqueducts, frequently without prior treatment (Duarte-Abadía & Boelens, 2016; Duarte-Abadía et al., 2023). Additionally, the Vetas River supplies water to the Bosconia water treatment plant, which provides freshwater to Bucaramanga, one of Colombia's most important cities, with over 1,200,000 inhabitants (Alonso et al., 2020; Coz et al., 2008).

The tap water samples generally had metal concentrations below the detection limit, except for Zn at the Vetas municipal administrative center, where it measured 0.02 mg/L. This concentration is well below the threshold levels associated with health risks, indicating that the water is safe for consumption according to guidelines established by the European Union (European Parliament, 2020) and Colombia (Ministry of Housing of Colombia, 2007).

### Pseudototal content of heavy metal in sediment samples

**Fig. 2:**
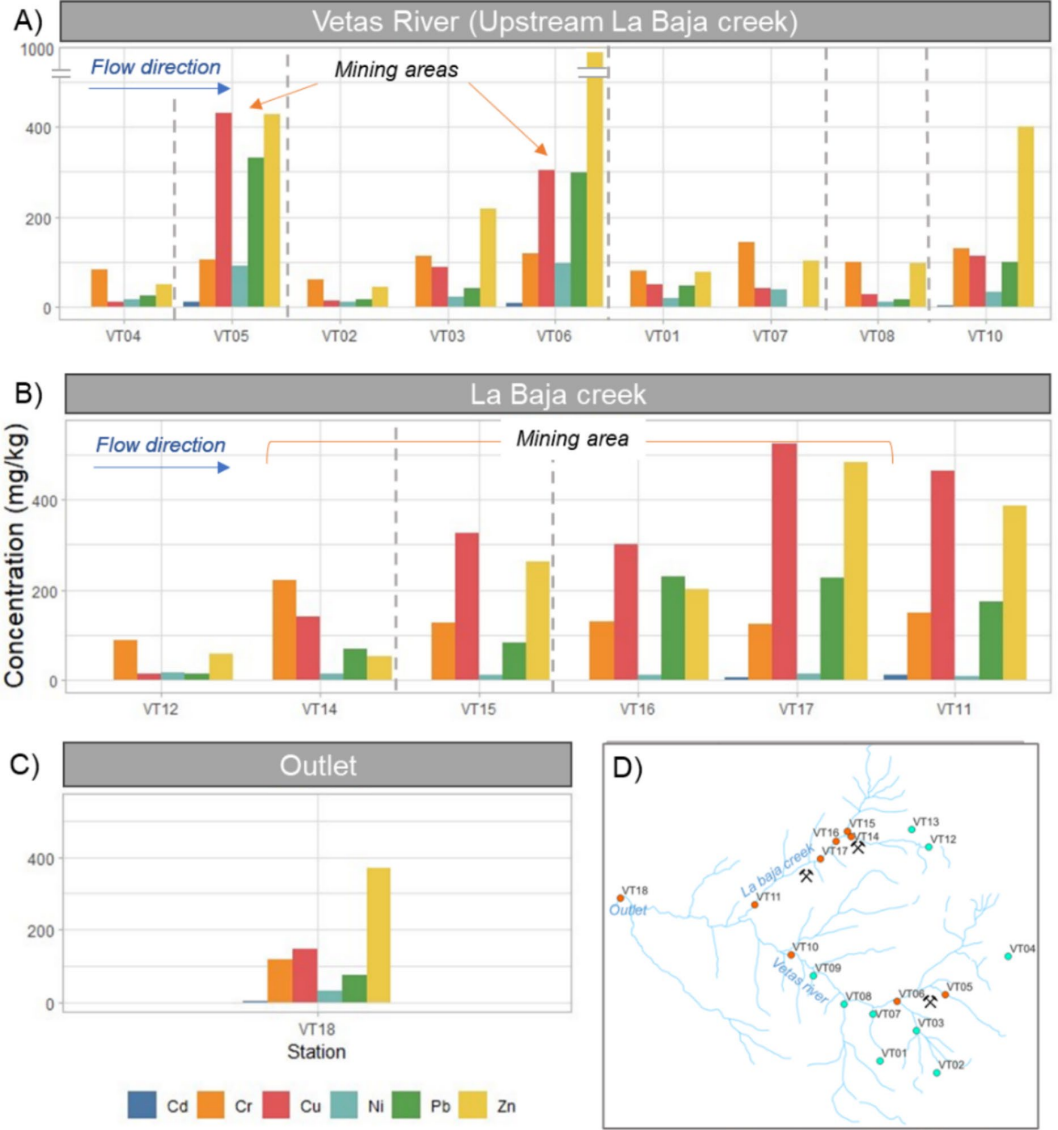
Concentration of Cd, Cr, Cu, Ni, Pb, and Zn in sediment samples of the Vetas River basin, Colombia. A) The Vetas River upstream of the confluence with La Baja Creek. B) La Baja Creek. C) The outlet of the catchment. D) Map with the location of the sampling points and mining areas. Dashed lines separate micro basins and empty columns indicated values below the detection limit

Pseudo-total heavy metal concentrations in sediments are presented in Fig. 2 and Table S5. The mean concentrations of metals followed the order: Zn (261.4 mg/kg) > Cu (187.3 mg/kg) > Cr (118.5 mg/kg) > Pb (109.7 mg/kg) > Ni (29.0 mg/kg) > Cd (3.1 mg/kg). The distribution of metal concentrations in sediments across sampling sites clearly correlated with mining activities. Higher concentrations were recorded in areas with active mining in El Volcán village (VT05 and VT06) and La Baja Creek (VT11, VT15-VT17), as can be seen in Fig. 2. At these points Zn, Cu and Pb displayed their highest values. Interestingly, VT14, located in La Baja Creek mining district, registered lower sediment concentrations than other mining areas, although the concentrations of many metals in water were one of the highest and exceeded drinking water guidelines set by the European Union Directive (European Parliament, 2020) and Colombian Resolution 2115 of 2007 (Ministry of Housing of Colombia, 2007). This can be explained because of a pH value in the site of only 2.78, the lowest recorded in the study area, and the increase in heavy metals solubility under acidic conditions. The values from the outlet showed lower concentrations compared to those from mining areas, suggesting a mixture of contaminated and uncontaminated sediments.

Results revealed a heterogeneous pattern in individual metal concentrations, with some metals increasing in mining areas while others remained unaffected. Al, Fe, Mn, and Ni were similar in both mining and non-mining areas. In contrast, Cr and chalcophile elements (Cd, Cu, Pb, and Zn) were generally above average in areas influenced by mining. Notably, Cd concentrations in non-mining areas were below the detection limit (Figure S2). These findings suggest that activities in mining areas may contribute to increased levels of sulfides and Cr in river sediments, altering the natural background concentrations.

#### Geoaccumulation index (Igeo)

**Fig. 3:**
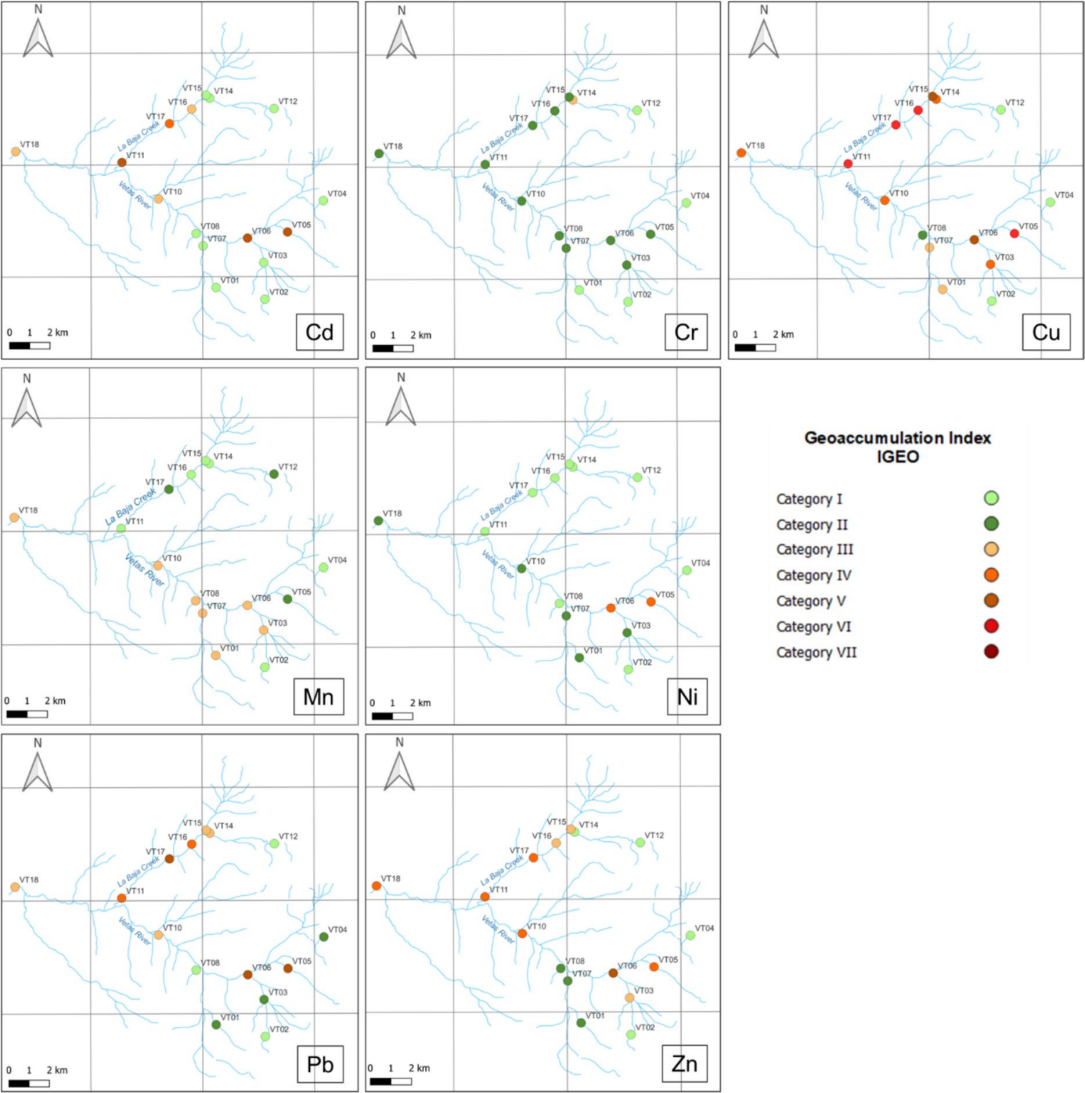
Spatial distribution of the geoaccumulation index in the Vetas River basin, Colombia, categorized according to Förstner and Müller (1981): category I (uncontaminated, ≤ 0), category II (uncontaminated to moderately contaminated, 0 < Igeo < 1), category III (moderately contaminated, 1 < Igeo < 2), category IV (moderately to strongly contaminated, 2 < Igeo < 3), category V (strongly contaminated, 3 < Igeo < 4), category VI (strongly to extremely contaminated, 4 < Igeo < 5) and category VII (extremely contaminated, Igeo ≥ 5)

Regarding the Geoaccumulation index (Igeo) and according to the classification proposed by Förstner and Müller (1981), sampling points in El Volcán village (VT05 and VT06) and La Baja Creek (VT11, VT15-VT17) presented metal contamination levels reaching strongly to extremely contaminated (Categories V and VI), as presented in Table S6. Cd, Cu, Pb and Zn had the highest Igeo values compared to Cr, Mn and Ni. Conversely, two sampling points located in the upper part of catchment, precisely in the Santurbán Páramo (VT04 and VT12), exhibited Igeo values primarily in categories I and II, indicating uncontaminated areas (Fig. 3 and Figure S3).

#### Pollution load index (PLI)

**Fig. 4:**
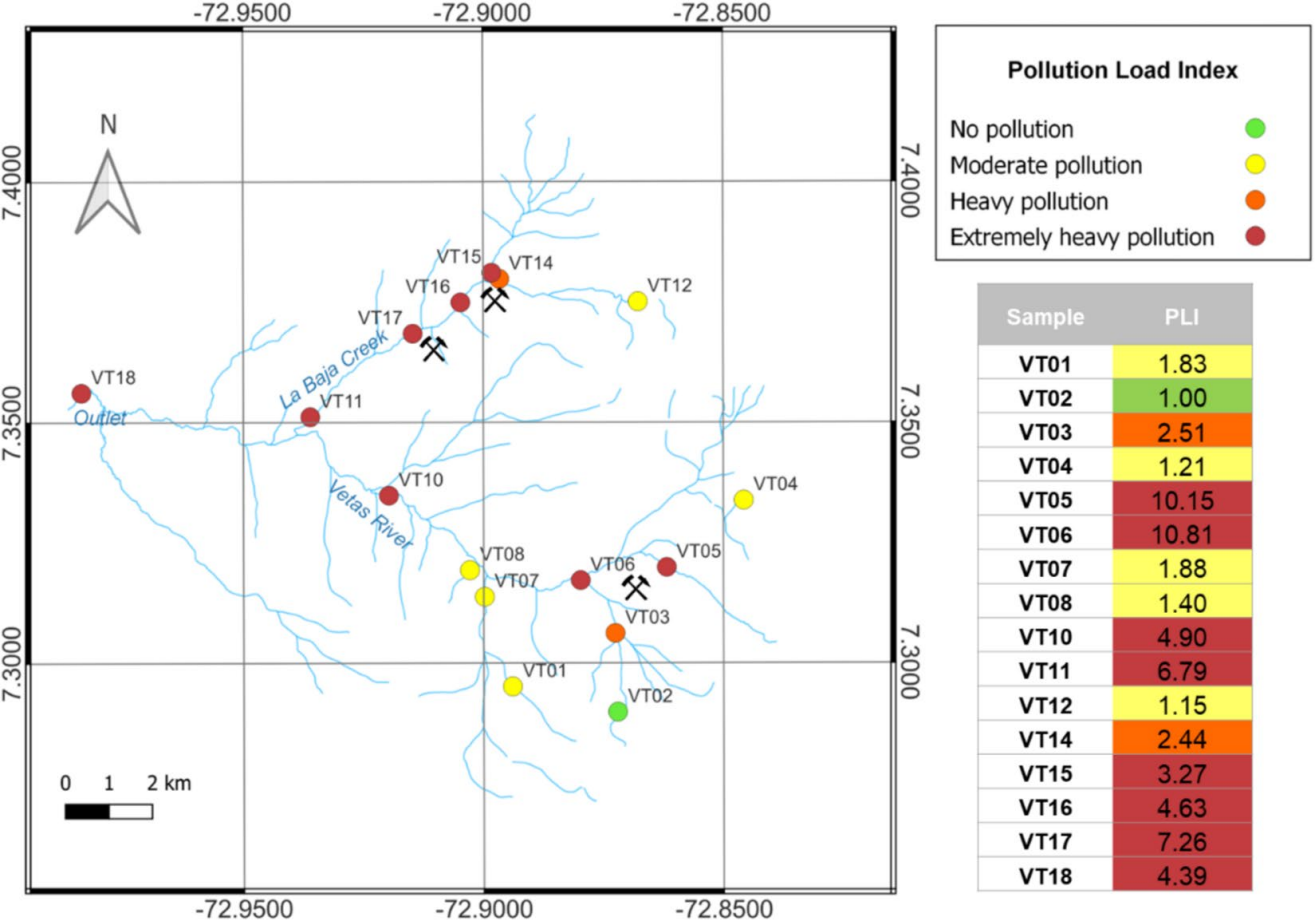
Pollution load index (PLI) in the Vetas River basin, Colombia, based on the contamination factor (CF) of Cd, Cr, Cu, Mn, Ni, Pb and Zn. The classification follows the criteria outlined by Aftab and Hakeem (2022) and Jorfi et al. (2017). Background value: VT02

The Pollution Load Index (PLI) ranged from 1.00 to 10.81 (Fig. 4). VT02 (upper part of the catchment) was the only site categorized as having no pollution. Therefore, this point served as the background reference. In contrast, the most severely affected areas were El Volcán Village and downstream (VT05-VT06), where the PLI exceeded 10, followed by La Baja Creek (VT15-VT17), the outlet (VT18), and the Vetas River before the confluence with la Baja Creek (VT10) and La Baja Creek before the confluence with the Vetas River. All of these sites were classified as having heavy to extremely heavy pollution. VT14 showed heavy pollution levels, a lower category than other points nearby, primarily due to its location and the low pH of the water, which keeps metals in solution. Whereas the points located in the Santurbán paramo exhibited moderate pollution levels.

These values are comparable to those found in other sulfide-rich mining areas that are unsuitable for cultivation (Fodoué et al., 2022; Liu et al., 2013). Fodoué et al. (2022), reported PLI values ranging from 5.3 to 19.9 in the surroundings of the Pawara gold mine in Cameroon, where a lack of regulation and control allows the use of illegal substances in artisanal mining. Liu et al. (2013) found PLI values between 1.34 and 5.14 in Jiangxi Province, China, where Au, Cu, Pb and Zn are extracted. The highest PLI values were recorded near mines and smelters. Similarly, in the vicinity of the Aznalcóllar mine in Spain, where pyrite and other sulfides are extracted, PLI values ranged from 2.0 to 8.4 despite the implementation of cleanup procedures following a mine tailing accident in 1998 (Galán et al., 2002).

#### Comparison to guidelines

The elevated metal concentrations in sediments found in the study area frequently exceeded the TEC threshold defined by MacDonald et al. (2000), indicating a possible toxicity effect. In the case of Cr, all the samples exceeded the TEC, which could indicate a high background level in the area. In some sampling points, even the PEC values were exceeded (Figure S4). In El Volcán village, the concentration of Cu, Ni, Pb and Zn at VT05 and Cr, Ni, Pb and Zn at VT06 exceeded the PEC. Similarly, concentrations of Cu, Cr, and Pb at VT16 and VT17 in La Baja Creek surpassed the PEC threshold, where mining is actively conducted. Although VT11 and VT18 are located outside the La Baja Creek mining district, they exhibited elevated concentrations of Cd, Cr, Cu, and Pb that exceeded the PEC threshold. This suggests a potential influence from upstream sampling points VT16 and VT17.

#### Statistical analysis: principal component analysis (PCA) and correlation analysis

The Kaiser–Meyer–Olkin (KMO) test yielded a value of 0.61, and the Bartlett's test produced a *p*-value below 0.05. These results confirm that the data are appropriate for PCA and correlation analysis. The first principal component (PC1) of the PCA analysis explained 64.9% of the total variance, while the second principal component (PC2) explained 24.5%. Together, they accounted for 89.4% of the total variance (Figure S5). The analysis revealed a positive correlation between Cd, Cr, Cu, Fe, Pb and Zn, which together explain the variance in PC1. Ni and Zn also displayed a positive correlation, although weaker, and explain the variance of PC2. In contrast, Al and Mn showed no correlations with the other elements. The plot of the two principal component (Figure S5) separates mining and non-mining areas in two different groups, with three points from non-mining areas in the Vetas River (VT03, VT07 y VT08) positioned closer to the mining affected cluster. PC1 primarily captures the overall impact of mining on sediment contamination, distinguishing between mining-affected and non-mining areas. PC2 reflects additional variability within mining-affected sites and within no mining-affected sites, highlighting the unique behavior of Ni and Zn due to localized geochemical or mining-related factors.

The Pearson correlation analysis indicated a very strong significant positive correlation (r > 0.8, *p* < 0.05) between Pb and Cu, as well as a strong significant positive correlation (r > 0.6, *p* < 0.05) between Zn/Cu, Zn/Pb, Zn/Ni, Fe/Cu, Ni/Pb, and Al/Mn (Figure S6). Those high correlation coefficient between metals may indicate similar behavior under similar environmental conditions and a common origin (Llorca et al., 2020). The Pb–Zn pair is typically indicative of anthropogenic pollution in industrial areas or mines (Bauer & Velde, 2014). The strong correlation among these elements (mainly chalcophiles) may be attributed to the presence and coexistence of sulfides with a shared origin, such as sphalerite (blende, (Zn,Fe)S), galena (PbS), bornite (Cu_5_FeS_4_), covellite (CuS), pyrite (FeS_2_), chalcopyrite (CuFeS_2_), chalcocite (Cu_2_S), among others, since they are formed under similar geologic processes (Fagbenro et al., 2021).

### Sequential extraction for bioavailable metals in sediments, bioavailable fraction toxicity factor and bioavailable fraction toxicity index

**Fig. 5:**
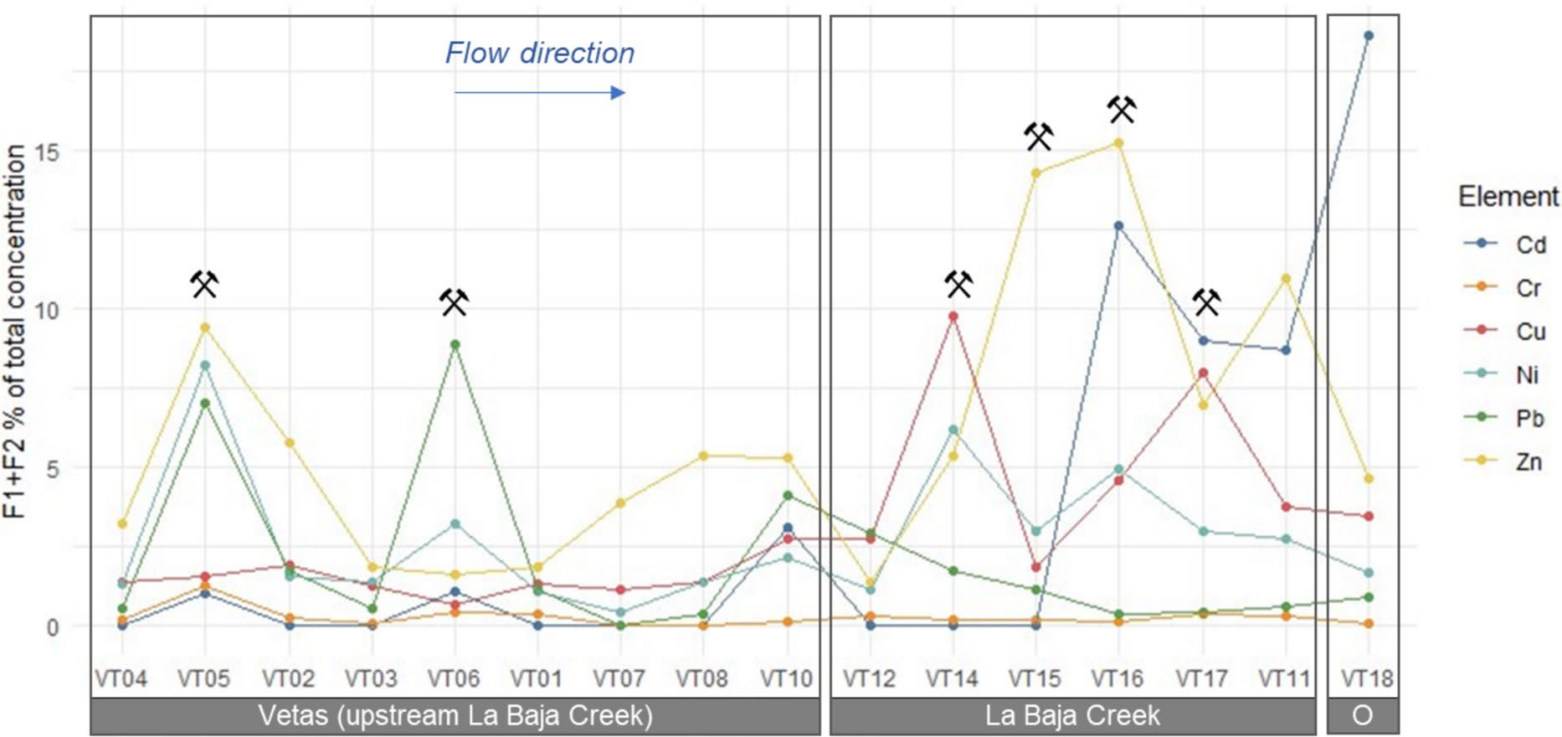
The percentage of the sum of Cd, Cr, Cu, Ni, Pb, and Zn concentrations in fraction 1 (exchangeable) and fraction 2 (bound to carbonates) relative to the pseudototal concentration in sediments of the Vetas River basin, Colombia. F1 + F2: sum fractions 1 and 2. O = Outlet

The percentage of the sum of metal concentrations in the exchangeable (F1) and bound to carbonates (F2) fractions relative to the pseudo-total fraction in sediments are presented in Fig. 5, Figure S7, Table S7, Table S8 and Table S9. The percentage were as follows: Cd (9.22%) > Zn (6.07%) > Pb (3.29%) > Cu (2.96%) > Ni (2.70%) > Cr (0.25%).

Cd, Cu, and Zn exhibited particularly elevated percentages in the mining areas of La Baja Creek, with average values of 8.0%, 5.6% and 10.6%, respectively, while remaining below 3% in non-mining areas of the creek. In the mining areas of the Vetas River, El Volcán village (VT05) showed the highest percentages, with Ni, Pb and Zn accounting for 7.0% to 9.4% of their total concentration. At VT06, Pb accounted for 8.9%. In non-mining locations, Zn exceeded 5% in VT02 and VT08, indicating either naturally high concentrations or contamination from other human activities such as agriculture and livestock (Ahmed et al., 2012).

Fractions 1 and 2 of the Tessier protocol are considered bioavailable fractions (Mittermüller et al., 2016). These results suggest that mining areas and downstream locations contain higher amounts and percentages of bioavailable metals compared to non-mining areas (Aguilar-Hinojosa et al., 2016), potentially increasing the risk of toxic effects on both the environment and human health. However, these results are lower than levels reported for Pb (14%) and Zn (21%) in the highly mining-impacted Tinto estuary (Rosado et al., 2016).

**Fig. 6:**
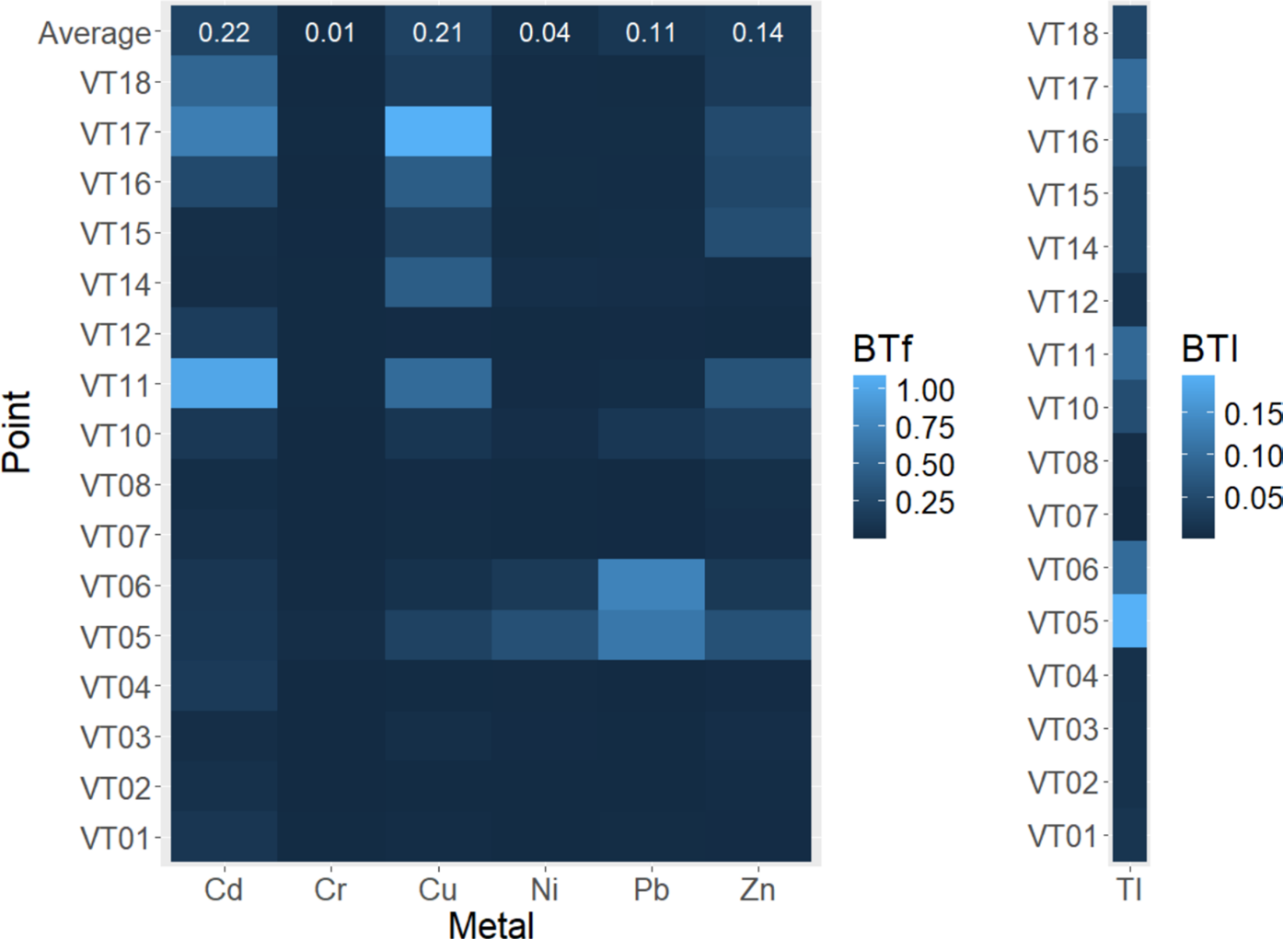
Bioavailable fraction toxicity factor (BTf) and bioavailable fraction toxicity index (BTI) in sediments of the Vetas River basin, Colombia

Figure 6 displays the average values of the bioavailable fraction toxicity factor (BTf) for each metal: Cd (0.22) > Cu (0.21) > Zn (0.14) > Pb (0.11) > Ni (0.04) > Cr (0.01). This indicates that, on average, Cd and Cu pose the highest toxicity risk to the aquatic ecosystem based on the sum of the exchangeable and bound to carbonates fractions, while Ni and Cr pose the lowest risk. On average, the sum of both fractions is below the threshold effect concentration (TEC), suggesting no expected toxicity risk to the aquatic environment when considering these fractions alone.

Based on the toxicity index values, the sampling points can be ranked as follows: VT07 (0.000) > VT08 (0.006) > VT04 (0.011) > VT02 (0.012) > VT03 (0.012) > VT12 (0.014) > VT01 (0.017) > VT14 (0.040) > VT15 (0.040) > VT18 (0.044) > VT10 (0.055) > VT16 (0.064) > VT11 (0.094) > VT06 (0.098) > VT17 (0.100) > VT05 (0.193), indicating an absence of toxicity risk when considering these fractions alone.

The PLI and BTI show a parallel relationship in this study, where higher pollution levels correlate with increased toxicity risk, underscoring the impact of pollution sources on toxicity. This is evident, for example, in VT05, which has the highest BTI and the second-highest PLI, and VT06, which ranks third in BTI and second in PLI.

## Conclusions

This study investigated heavy metal pollution (Cd, Cr, Cu, Fe, Mn, Ni, Pb, and Zn) in the Vetas river catchment to evaluate their potential toxicity risks to biota, with a focus on the influence of artisanal and small-scale mining activities. The findings confirmed the hypothesis that mining areas are strongly associated with elevated concentrations of heavy metals in both water and sediments, particularly in the La Baja Creek and El Volcán Village districts. These areas were identified as contamination hotspots, with pseudototal metal concentrations in sediments categorized as strongly to strongly-to-extremely contaminated based on the geoaccumulation index (Igeo) and heavily to extremely heavily polluted based on the pollution load index (PLI). In contrast, the Santurbán Páramo exhibited moderate pollution levels. Cu, Pb and Zn were the most enriched metals, reaching strongly to extremely contaminated levels in mining areas. Additionally, the concentrations of Cr, Cu, Ni, Pb and Zn in these areas exceeded the Probable Effect Concentration (PEC) threshold, indicating a toxicity risk to biota.

The application of the Tessier sequential extraction method revealed elevated concentrations of bioavailable fractions in mining districts and downstream areas compared to non-mining zones. These findings suggest a higher potential for heavy metal release under acidic conditions in mining and downstream regions. Furthermore, represents the first dataset on the bioavailable fractions of heavy metals in sediments from the Vetas River catchment.

The development of the Bioavailable Fraction Toxicity Factor (BTf) and the Bioavailable Fraction Toxicity Index (BTI) offers novel tools to assess heavy metal toxicity risks in sediments. These indices are based on the bioavailable fractions of heavy metals, specifically the exchangeable and carbonate-bound fractions, providing a more nuanced understanding of metal toxicity risks compared to traditional approaches that rely solely on pseudototal concentrations. While some sites showed low toxicity risk when considering bioavailable fractions alone, those with significant mining influence exhibited higher BTI values. These indices are promising tools for environmental monitoring and provide a clearer picture of the ecological impact of metal pollution.

Given the ecological and hydrological significance of the Santurbán páramo, a component of the Tropical Andes biodiversity hotspot and a key freshwater source for the city of Bucaramanga, there is an urgent need for measures to mitigate heavy metal pollution and implement effective remediation techniques. Considering the high biodiversity of Colombia, further research is needed to explore the remediation potential of plants in the study area, providing a foundation for future sustainable management practices.

## Supplementary Information

Below is the link to the electronic supplementary material.

Supplementary file1 (DOCX 2134 kb)

## Data Availability

The datasets generated during and/or analysed during the current study are available from the corresponding author on reasonable request.
