# Supplementary material for: Assessing sediment toxicity risks with bioavailable metal fractions: new factors and index applied to the Colombian tropical Andes hotspot
Source: Environ Geochem Health. 2025 May 22;47(6):222. doi: 10.1007/s10653-025-02536-3 (PMC12098199; doi:10.1007/s10653-025-02536-3)
Supplement: Supplementary file 1 — Supplementary file1 (DOCX 2134 kb) [file 10653_2025_2536_MOESM1_ESM.docx]

# Supplementary material

Table S1. Coordinates of the sampling points.

| Point | North | West |
| --- | --- | --- |
| VT01 | 7°17'42.5" | 72°53'38.3" |
| VT02 | 7°17'23.5" | 72°52'19.4" |
| VT03 | 7°18'22.4" | 72°52'21.3" |
| VT04 | 7°20'01.8" | 72°50'44.8" |
| VT05 | 7°19'11.6" | 72°51'42.6" |
| VT06 | 7°19'01.9" | 72°52'47.2" |
| VT07 | 7°18'49.6" | 72°53'59.2" |
| VT08 | 7°19'09.5" | 72°54'10.3" |
| VT09 | 7°19'37.0" | 72°54'40.4" |
| VT10 | 7°20'05.4" | 72°55'10.9" |
| VT11 | 7°21'04.1" | 72°56'09.8" |
| VT12 | 7°22'37.5" | 72°52'07.6" |
| VT13 | 7°22'53.8" | 72°52'27.3" |
| VT14 | 7°22'47.3" | 72°53'47.8" |
| VT15 | 7°22'51.9" | 72°53'53.7" |
| VT16 | 7°22'29.7" | 72°54'17.0" |
| VT17 | 7°22'06.4" | 72°54'52.9" |
| VT18 | 7°21'22.1" | 72°59'01.5" |

Table S2. Reagents and conditions of the sequential extraction procedure proposed by Tessier et al. (1979).

| Fraction | Leach reagents | Concentration | Volume/1g Sample | pH | Temperature | Shaking | Extraction time |
| --- | --- | --- | --- | --- | --- | --- | --- |
| Exchangeable | MgCl_2_ | 1M | 8ml | 7 | Room | Constant | 1h |
| Carbonates | NaOAc | 1M | 8ml | 5 (HOAc) | Room | Constant | 2h |

Table S3. In situ water quality parameters in the Vetas River catchment, Colombia.

| Point | pH | Conductivity (µS/cm) | Dissolved oxygen (mg/L) | Temperature (^o^C) | |
| --- | --- | --- | --- | --- | --- |
| VT00 | 7.27 | 149 | 7.47 | 11.7 | |
| VT01 | 7.64 | 44 | 7.68 | 9.5 | |
| VT02 | 7.50 | 25 | 7.97 | 12.2 | |
| VT03 | 7.47 | 69 | 7.86 | 9.1 | |
| VT04 | 7.34 | 24 | 7.18 | 13.9 | |
| VT05 | 4.47 | 350 | 7.60 | 10.8 | |
| VT06 | 6.89 | 405 | 8.14 | 10.2 | |
| VT07 | 7.53 | 43 | 8.63 | 9.4 | |
| VT08 | 7.38 | 59 | 7.84 | 11.2 | |
| VT09 | 7.13 | 74 | 7.38 | 13.7 | |
| VT10 | 7.40 | 141 | 8.50 | 12.3 | |
| VT11 | 7.89 | 426 | 8.04 | 15.0 | |
| VT12 | 7.83 | 69 | 7.49 | 9.7 | |
| VT13 | 7.86 | 70 | 7.34 | 9.5 | |
| VT14 | 2.78 | 1787 | 7.27 | 15.5 | |
| VT15 | 6.84 | 72 | 8.17 | 11.4 | |
| VT16 | 6.88 | 314 | 7.80 | 14.0 | |
| VT17 | 5.03 | 377 | 7.82 | 14.9 | |
| VT18 | 7.69 | 238 | 8.01 | 18.0 | |
| VT19 | 7.09 | 158 | 7.52 | 11.7 | |
| Range | 2.78-7.89 | 24.2-1787 | 7.18-8.63 | 9.08–18.00 | |
| Median | 7.39 | 72.6 | 7.83 | 11.80 | |
| Mean | 6.86 | 254.8 | 7.82 | 12.24 | |
| Standard deviation | 1.37 | 409.1 | 0.40 | 2.59 | |
| Municipal centres | 1.37 |  |  | |  |
| Vetas | 7.27 | 149.0 | 7.47 | 11.7 | |
| California | 7.09 | 158.0 | 7.52 | 11.7 | |
| Regulations |  |  |  |  | |
| Drinking water guidelines  Directive (EU) 2020/2184 | 6.5-9.5 | 2500 |  |  | |
| Colombian drinking-water regulations  Resolución 2115 of 2007 | 6.5-9.0 | 1000 |  |  | |
| Colombian discharge into water bodies regulations (mining waters)  Resolución 631 of 2015 | 6.0-9.0 |  |  |  | |
| Directive on the quality of fresh waters needing protection or improvement to support fish life  Council Directive 78/659/EEC |  |  | Salmonids  Obligatory: >6  Cyprinidae  Obligatory: >4 |  | |


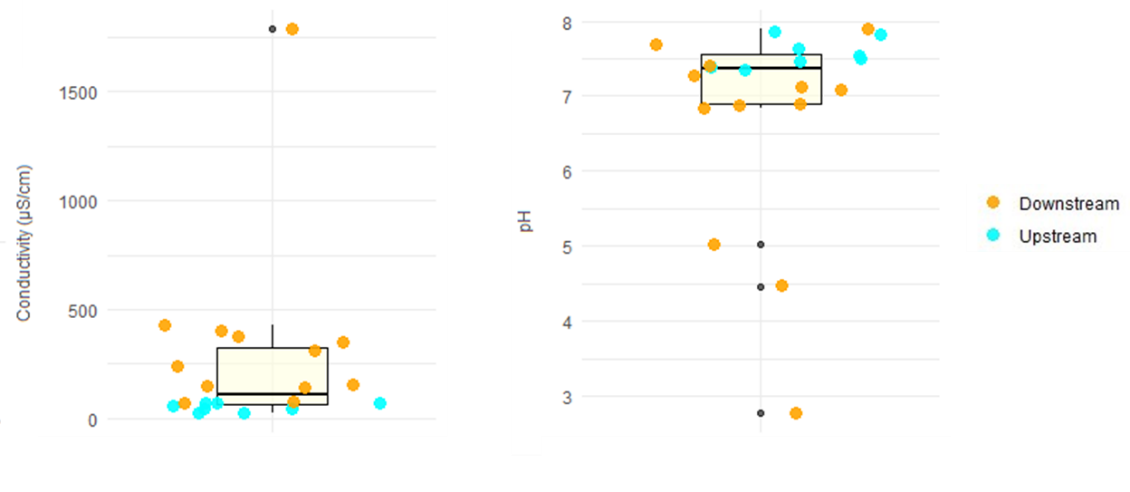


Figure S1. pH and conductivity distribution in the Vetas River basin, Colombia. Blue dots represent sampling points located upstream or within micro basins without mining activities (VT01 - VT04, VT07, VT08, VT12, and VT13) and orange dots represent sampling points in mining districts or downstream areas (VT05, VT06, VT11, VT14 - VT18).

Table S4. Pseudo-total fraction, contamination factors (Cfs) and pollution load index (PLI) in the sediments of the Vetas River catchment, Colombia.

| Station | Cd  (mg/L) | Cr (mg/L) | Cu (mg/L) | Fe (mg/L) | Mn (mg/L) | Ni (mg/L) | Pb  (mg/L) | Zn (mg/L) | Reference |
| --- | --- | --- | --- | --- | --- | --- | --- | --- | --- |
| Vetas river, Colombia | 0.0653  0.0006-0.13 | 0.09  0.05-0.13 | 3.13  0.01-9.36 | 10.95  0.01-116.4 | 5.00  0.11-22.85 | 1.72  0.03-7.35 | 1.75  0.08-3.12 | 7.59  0.01-72.94 | This study |
| Odiel river, Spain | 0.081  0.001-0.380 |  | 7.60  0.01-37.62 | 23.47  0.03-262.71 | 13.70  0.49-60.33 |  | 0.207  0.004-  1.180 | 24.23  0.17-130.23 | (Olías et al., 2004) |
| Escalera River, Peru | 0.17  0.109-  0.225 |  |  | 99.13  2.48-  234 | 6.75  5.14-7.87 |  | 0.74  0.45-0.95 | 37.02  23.89-54.86 | (Cacciuttolo and Cano, 2022) |
| Kor River, Iran | 6.12 0.13–17.36 | 20.63  0.74–55.50 | 8.95  0.12–22.71 |  |  | 7.63  1.10–25.28 |  | 37.93  21.92–225.17 | (Mokarram et al., 2020) |
| Wenyu River, Qinling, China | 22.430  0.09-  66.17 | 1.039  0.11-  5.59 | 1.083  0.01-  6.55 |  | 2.818  0.01-  15.84 | 37.250  1.84-  141.4 | 1.696  0.74-  3.98 | 2.514  0.01-  16.16 | (Chen et al., 2021) |

Table S5. Pseudo-total fraction, contamination factors (Cfs) and pollution load index (PLI) in the sediments of the Vetas River catchment, Colombia.

|  |  | Fe | Mn | Cd | Cr | Cu | Ni | Pb | Zn |
| --- | --- | --- | --- | --- | --- | --- | --- | --- | --- |
| Pseudo-total fraction (mg/kg) | VT01 | 27221 | 1032 | 0.68 | 80.8 | 51.2 | 21.0 | 46.8 | 78.2 |
|  | VT02 | 17058 | 328 | 0.68 | 60.8 | 13.5 | 13.3 | 16.9 | 46.0 |
|  | VT03 | 38847 | 1421 | 0.68 | 114.4 | 89.0 | 22.7 | 41.9 | 219.3 |
|  | VT04 | 24768 | 471 | 0.68 | 83.8 | 13.3 | 18.8 | 25.7 | 50.4 |
|  | VT05 | 42062 | 513 | 10.80 | 105.3 | 429.9 | 91.4 | 331.8 | 428.1 |
|  | VT06 | 37512 | 1843 | 9.30 | 119.2 | 302.9 | 97.5 | 299.7 | 940.3 |
|  | VT07 | 37157 | 1269 | 0.68 | 142.9 | 43.4 | 38.5 | 0.9 | 103.4 |
|  | VT08 | 37600 | 1354 | 0.68 | 99.8 | 27.9 | 12.4 | 18.7 | 97.9 |
|  | VT09 |  |  |  |  |  |  |  |  |
|  | VT10 | 33192 | 1523 | 3.90 | 130.2 | 114.6 | 35.2 | 99.0 | 399.8 |
|  | VT11 | 44278 | 183 | 11.70 | 150.3 | 463.5 | 10.4 | 173.5 | 387.6 |
|  | VT12 | 26748 | 836 | 0.68 | 88.6 | 14.0 | 17.6 | 15.2 | 59.5 |
|  | VT13 |  |  |  |  |  |  |  |  |
|  | VT14 | 32626 | 94 | 0.68 | 221.6 | 141.1 | 15.0 | 69.3 | 54.7 |
|  | VT15 | 50200 | 101 | 0.68 | 128.3 | 324.5 | 11.2 | 83.6 | 263.6 |
|  | VT16 | 38100 | 181 | 2.13 | 129.7 | 300.0 | 11.7 | 229.7 | 201.6 |
|  | VT17 | 37213 | 611 | 7.70 | 124.3 | 523.2 | 15.5 | 226.1 | 483.0 |
|  | VT18 | 36382 | 990 | 2.70 | 116.1 | 145.4 | 32.2 | 75.8 | 369.6 |
| Average |  | 35060 | 797 | 3.40 | 118.5 | 187.3 | 29.0 | 109.7 | 261.4 |
| Contamination factor (Cf) | VT01 |  | 3.15 | 1.00 | 1.33 | 3.79 | 1.58 | 2.77 | 1.70 |
|  | VT02 |  | 1.00 | 1.00 | 1.00 | 1.00 | 1.00 | 1.00 | 1.00 |
|  | VT03 |  | 4.33 | 1.00 | 1.88 | 6.59 | 1.71 | 2.48 | 4.77 |
|  | VT04 |  | 1.44 | 1.00 | 1.38 | 0.99 | 1.41 | 1.52 | 1.10 |
|  | VT05 |  | 1.56 | 15.88 | 1.73 | 31.84 | 6.87 | 19.63 | 9.31 |
|  | VT06 |  | 5.62 | 13.68 | 1.96 | 22.44 | 7.33 | 17.73 | 20.44 |
|  | VT07 |  | 3.87 | 1.00 | 2.35 | 3.21 | 2.89 | 0.90 | 2.25 |
|  | VT08 |  | 4.13 | 1.00 | 1.64 | 2.07 | 0.93 | 1.11 | 2.13 |
|  | VT09 |  |  |  |  |  |  |  |  |
|  | VT10 |  | 4.64 | 5.74 | 2.14 | 8.49 | 2.65 | 5.86 | 8.69 |
|  | VT11 |  | 0.56 | 17.21 | 2.47 | 34.33 | 0.78 | 10.27 | 8.43 |
|  | VT12 |  | 2.55 | 1.00 | 1.46 | 1.04 | 1.32 | 0.90 | 1.29 |
|  | VT13 |  |  |  |  |  |  |  |  |
|  | VT14 |  | 0.29 | 1.00 | 3.64 | 10.45 | 1.13 | 4.10 | 1.19 |
|  | VT15 |  | 0.31 | 1.00 | 2.11 | 24.04 | 0.84 | 4.95 | 5.73 |
|  | VT16 |  | 0.55 | 3.13 | 2.13 | 22.22 | 0.88 | 13.59 | 4.38 |
|  | VT17 |  | 1.86 | 11.32 | 2.04 | 38.76 | 1.17 | 13.38 | 10.50 |
|  | VT18 |  | 3.02 | 3.97 | 1.91 | 10.77 | 2.42 | 4.49 | 8.03 |
| Average |  |  | 2.43 | 5.00 | 1.95 | 13.88 | 2.18 | 6.54 | 5.68 |
| Pollution load index (PLI) | VT01 | VT02 | VT03 | VT04 | VT05 | VT06 | VT07 | VT08 | VT09 |
|  | 1.83 | 1.00 | 2.51 | 1.12 | 10.16 | 10.81 | 1.88 | 1.40 |  |
|  | VT10 | VT11 | VT12 | VT13 | VT14 | VT15 | VT16 | VT17 | VT18 |
|  | 4.91 | 6.80 | 1.15 |  | 2.44 | 3.26 | 4.45 | 7.26 | 4.39 |


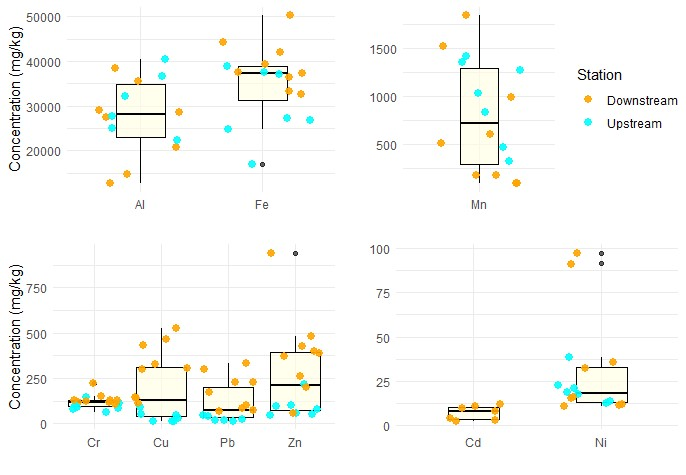


Figure S2. Distribution of heavy metals in sediments of the Vetas River basin, Colombia. Blue dots represent sampling points located upstream mining areas or micro basins without mining activities. Orange dots represent areas within or downstream mining districts.

Table S6. Geoaccumulation index (Igeo) of samples from the river Vetas. Colombia. Categorization according to Förstner and Müller (1981).

| Sample | Cd | Cr | Cu | Mn | Ni | Pb | Zn | Avergae |
| --- | --- | --- | --- | --- | --- | --- | --- | --- |
| VT01 | -0.58 | -0.18 | 1.34 | 1.07 | 0.07 | 0.88 | 0.18 | 0.40 |
| VT02* | -0.58 | -0.58 | -0.58 | -0.58 | -0.58 | -0.58 | -0.58 | -0.58 |
| VT03 | -0.58 | 0.33 | 2.13 | 1.53 | 0.19 | 0.72 | 1.67 | 0.86 |
| VT04 | -0.58 | -0.12 | -0.61 | -0.06 | -0.09 | 0.02 | -0.45 | -0.27 |
| VT05 | 3.40 | 0.21 | 4.41 | 0.06 | 2.19 | 3.71 | 2.63 | 2.37 |
| VT06 | 3.19 | 0.39 | 3.90 | 1.91 | 2.29 | 3.56 | 3.77 | 2.71 |
| VT07 | -0.58 | 0.65 | 1.10 | 1.37 | 0.94 | -0.45 | 0.58 | -0.11 |
| VT08 | -0.58 | 0.13 | 0.46 | 1.46 | -0.68 | -0.43 | 0.50 | 0.12 |
| VT10 | 1.92 | 0.51 | 2.50 | 1.63 | 0.82 | 1.97 | 2.53 | 1.70 |
| VT11 | 3.52 | 0.72 | 4.52 | -1.43 | -0.94 | 2.78 | 2.49 | 1.66 |
| VT12 | -0.58 | -0.04 | -0.54 | 0.76 | -0.18 | -0.74 | -0.21 | -0.22 |
| VT14 | -0.58 | 1.28 | 2.80 | -2.38 | -0.41 | 1.45 | -0.34 | 0.26 |
| VT15 | -0.58 | 0.49 | 4.00 | -2.29 | -0.83 | 1.72 | 1.93 | 0.63 |
| VT16 | 1.26 | 0.62 | 4.04 | -1.44 | -0.71 | 2.94 | 1.62 | 1.14 |
| VT17 | 2.92 | 0.45 | 4.69 | 0.31 | -0.37 | 3.16 | 2.81 | 2.00 |
| VT18 | 1.41 | 0.35 | 2.84 | 1.01 | 0.69 | 1.58 | 2.42 | 1.47 |
| Average | 0.76 | 0.32 | 2.30 | 0.18 | 0.15 | 1.13 | 1.34 |  |


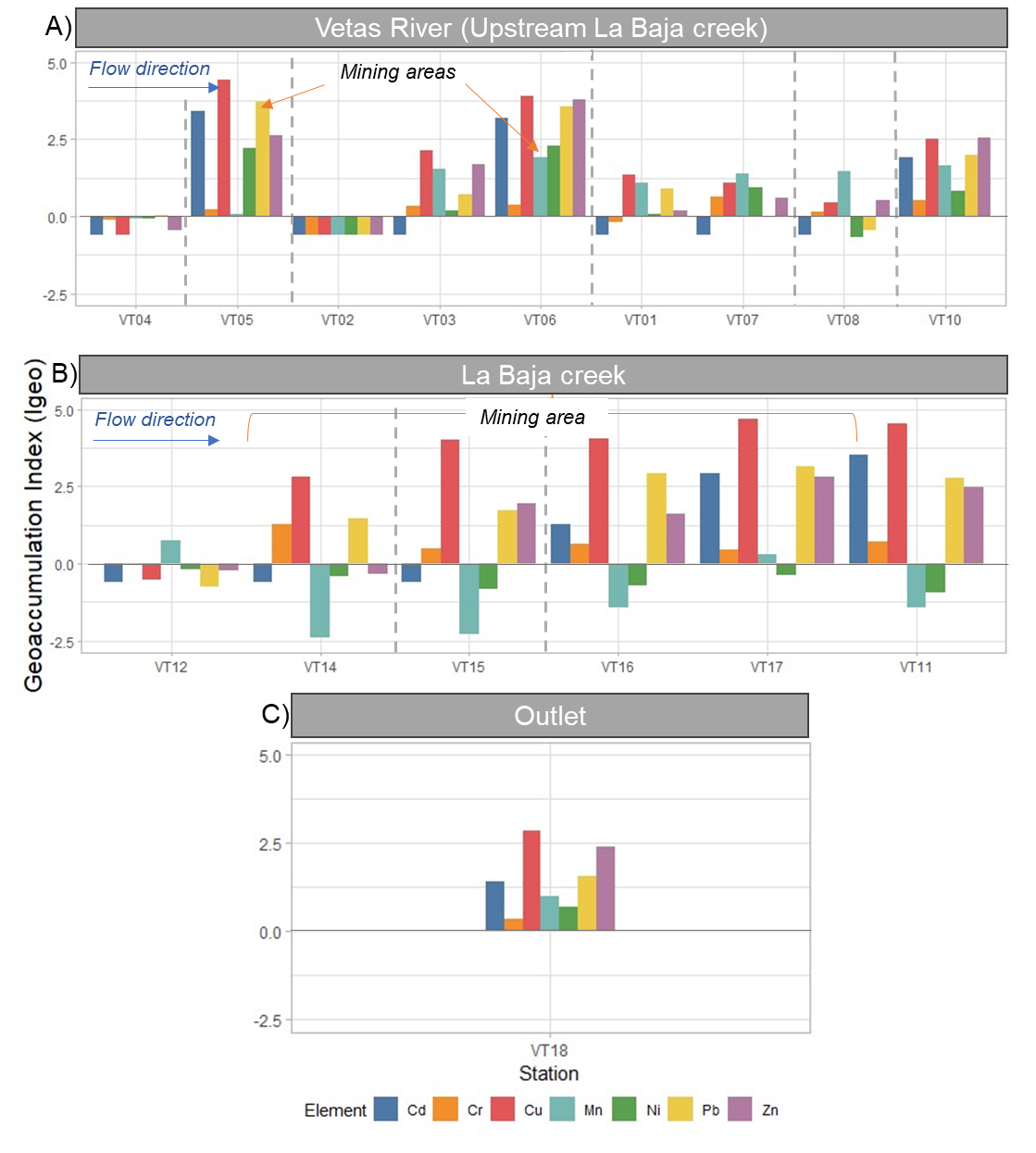


Figure S3. Geoaccumulation index of Cd, Cr, Cu, Mn, Ni, Pb and Zn in sediment samples of the Vetas River basin, Colombia. A) The Vetas River upstream of the confluence with La Baja Creek. B) La Baja Creek. C) The outlet of the catchment. Dashed lines indicated independent micro basins.


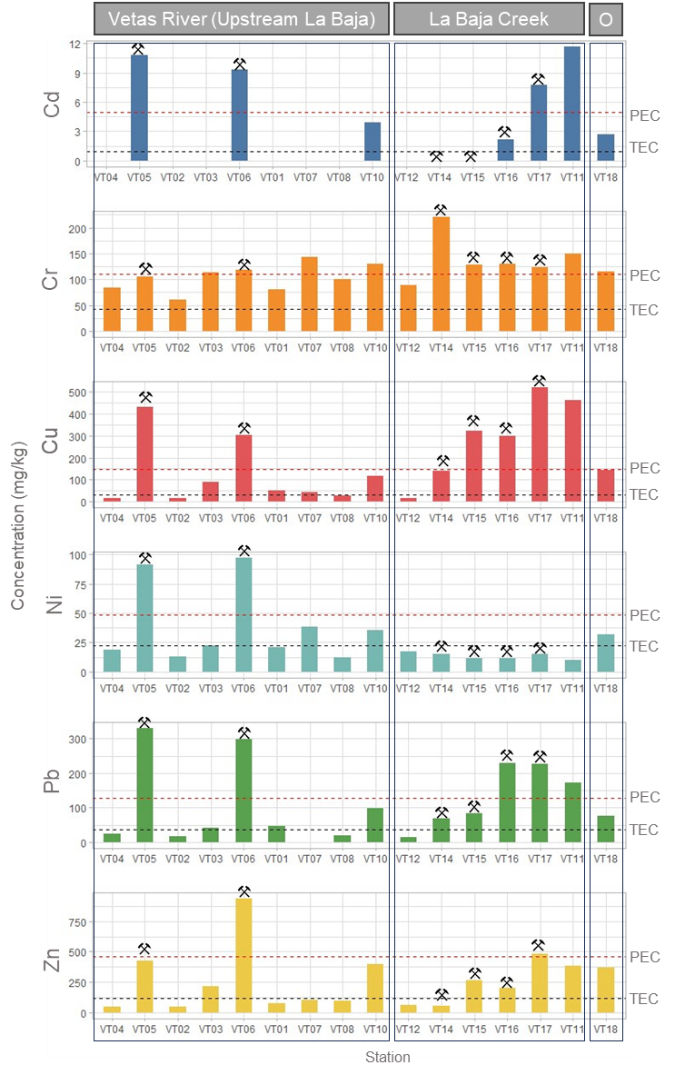


Figure S4. Comparison between pseudototal concentration of heavy metals in sediments and Threshold Effect Concentrations (TEC, black dashed line) and Probable Effect Concentration (PEC, red dashed line) thresholds (MacDonald et al., 2000) in the Vetas River basin, Colombia. O= Outlet.


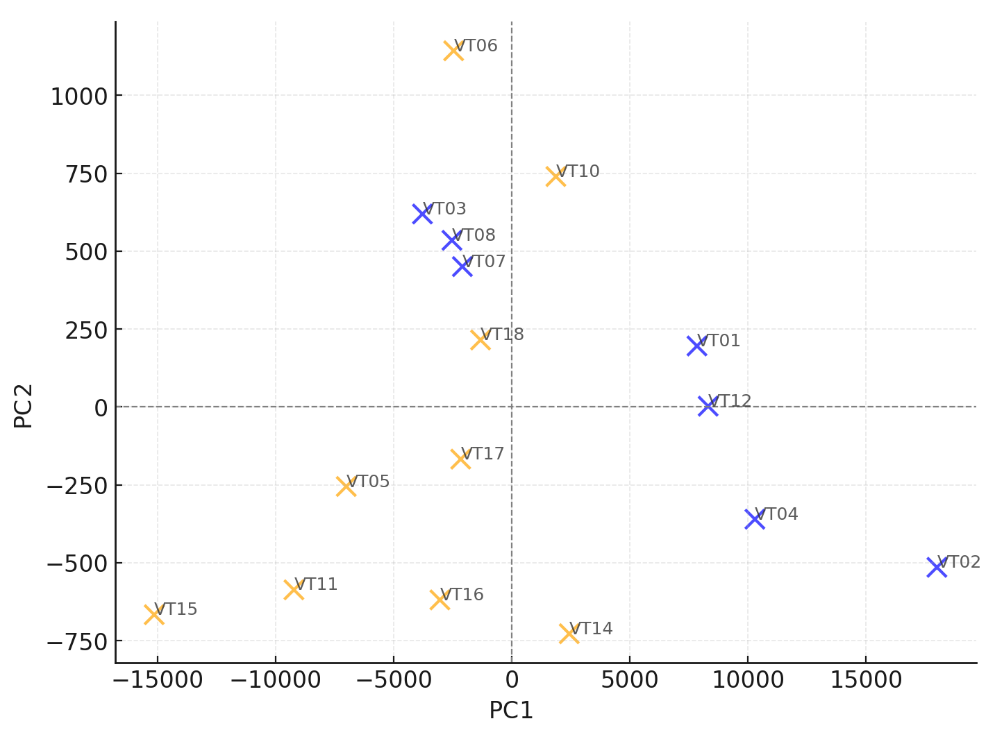


Figure S5. Principal component analysis (PCA) of the pseudototal concentrations of metals in sediments of the Vetas River basin, Colombia. Blue dots: sampling points located upstream mining areas or in micro basins without mining activities. Orange dots: areas within or downstream mining districts.


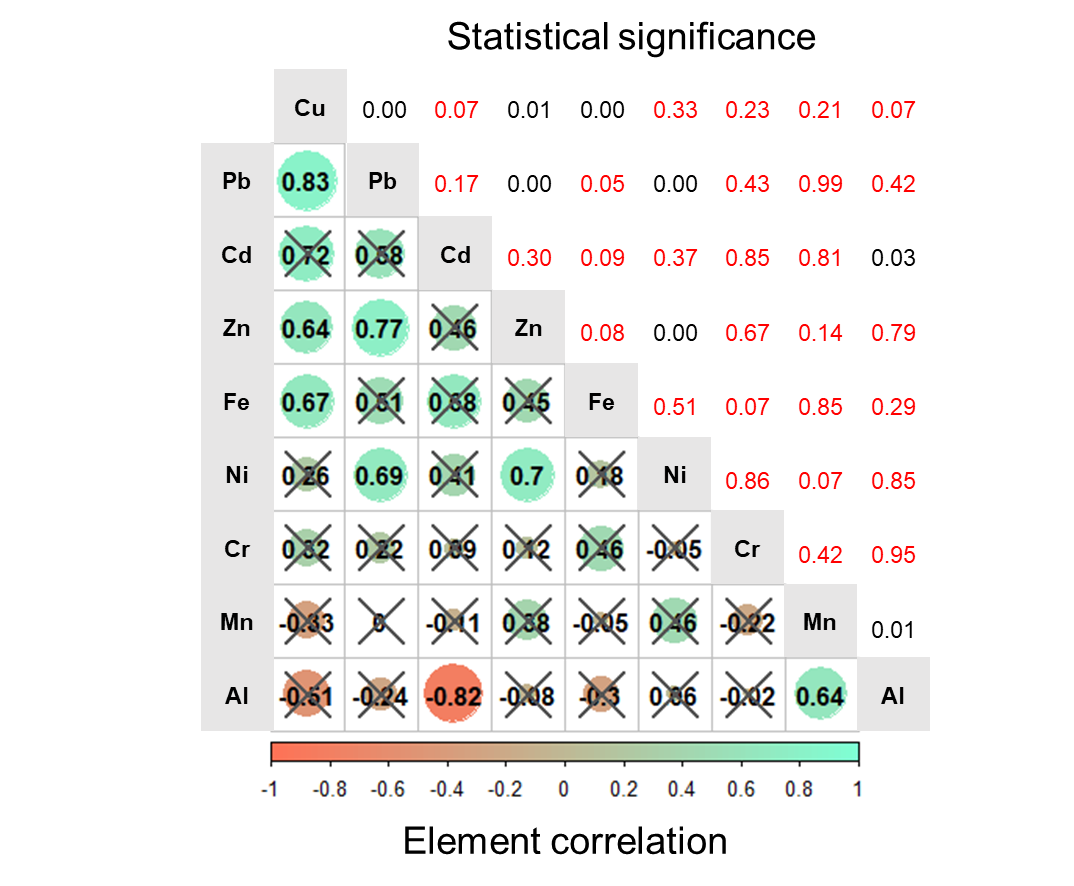


Figure S6. Pearson correlation matrix and statistical significance values of the pseudototal concentrations of metals in sediments of the Vetas River basin, Colombia. X and red values highlight non-significant correlations (p values > 0.05).

Table S7. Concentrations of metals in exchangeable (F1) and bound to carbonates (F2) fractions in the sediments of Vetas river catchment, Colombia according to the sequential extraction procedure proposed by Tessier et al. (1979).

|  |  | Fe | Mn | Cd | Cr | Cu | Ni | Pb | Zn |
| --- | --- | --- | --- | --- | --- | --- | --- | --- | --- |
| Exchangeable  (mg/kg) | VT01 | 1.47 | 16.18 | 0.091 | 0.039 | 0.115 | 0.191 | 0.085 | 1.090 |
|  | VT02 | 2.94 | 12.80 | 0.055 | 0.027 | 0.102 | 0.211 | 0.169 | 2.558 |
|  | VT03 | 0.67 | 20.28 | 0.029 | 0.000 | 0.098 | 0.209 | 0.027 | 2.270 |
|  | VT04 | 5.96 | 120.25 | 0.131 | 0.002 | 0.046 | 0.234 | 0.082 | 1.528 |
|  | VT05 | 137.48 | 95.23 | 0.113 | 0.896 | 5.303 | 7.370 | 21.953 | 40.000 |
|  | VT06 | 57.70 | 59.55 | 0.102 | 0.006 | 0.941 | 2.965 | 12.570 | 13.553 |
|  | VT07 | 0.84 | 20.87 | 0.049 | 0.000 | 0.111 | 0.096 | 0.014 | 2.132 |
|  | VT08 | 0.83 | 32.15 | 0.026 | 0.000 | 0.073 | 0.066 | 0.000 | 3.343 |
|  | VT09 |  |  |  |  |  |  |  |  |
|  | VT10 | 2.35 | 44.13 | 0.098 | 0.004 | 0.325 | 0.304 | 0.316 | 9.378 |
|  | VT11 | 3.44 | 14.91 | 0.636 | 0.009 | 3.010 | 0.099 | 0.115 | 14.675 |
|  | VT12 | 1.36 | 2.34 | 0.112 | 0.129 | 0.183 | 0.145 | 0.135 | 0.372 |
|  | VT13 |  |  |  |  |  |  |  |  |
|  | VT14 | 72.95 | 8.75 | 0.031 | 0.268 | 12.688 | 0.875 | 0.948 | 2.905 |
|  | VT15 | 2.72 | 13.01 | 0.036 | 0.000 | 1.640 | 0.241 | 0.295 | 27.650 |
|  | VT16 | 0.57 | 44.38 | 0.253 | 0.000 | 4.708 | 0.455 | 0.260 | 24.413 |
|  | VT17 | 0.80 | 26.08 | 0.407 | 0.000 | 0.425 | 0.084 | 0.000 | 1.984 |
|  | VT18 | 0.89 | 11.38 | 0.392 | 0.001 | 0.245 | 0.226 | 0.028 | 4.585 |
| Average |  | 18.31 | 33.89 | 0.160 | 0.086 | 1.876 | 0.861 | 2.312 | 9.527 |
| Bound to Carbonates (mg/kg) | VT01 | 7.74 | 8.44 | 0.01 | 0.23 | 0.55 | 0.04 | 0.45 | 0.36 |
|  | VT02 | 15.16 | 2.32 | 0.00 | 0.12 | 0.16 | 0.00 | 0.12 | 0.11 |
|  | VT03 | 19.85 | 18.51 | 0.00 | 0.04 | 1.01 | 0.10 | 0.20 | 1.71 |
|  | VT04 | 89.33 | 18.76 | 0.01 | 0.15 | 0.14 | 0.01 | 0.05 | 0.09 |
|  | VT05 | 22.53 | 2.93 | 0.00 | 0.42 | 1.26 | 0.13 | 1.33 | 0.42 |
|  | VT06 | 60.49 | 4.69 | 0.00 | 0.46 | 1.02 | 0.20 | 13.99 | 1.59 |
|  | VT07 | 4.86 | 34.57 | 0.00 | 0.00 | 0.39 | 0.06 | 0.17 | 1.88 |
|  | VT08 | 14.24 | 38.38 | 0.00 | 0.01 | 0.30 | 0.10 | 0.10 | 1.88 |
|  | VT09 |  |  |  |  |  |  |  |  |
|  | VT10 | 43.68 | 14.17 | 0.02 | 0.14 | 2.84 | 0.44 | 3.77 | 11.73 |
|  | VT11 | 81.14 | 14.84 | 0.38 | 0.44 | 14.45 | 0.19 | 0.87 | 27.74 |
|  | VT12 | 5.32 | 12.73 | 0.05 | 0.13 | 0.20 | 0.05 | 0.31 | 0.43 |
|  | VT13 |  |  |  |  |  |  |  |  |
|  | VT14 | 42.58 | 0.35 | 0.00 | 0.14 | 1.08 | 0.05 | 0.23 | 0.04 |
|  | VT15 | 15.06 | 3.02 | 0.00 | 0.23 | 4.33 | 0.10 | 0.65 | 10.03 |
|  | VT16 | 32.89 | 3.34 | 0.02 | 0.18 | 8.99 | 0.12 | 0.55 | 6.35 |
|  | VT17 | 41.78 | 61.93 | 0.29 | 0.41 | 41.38 | 0.38 | 0.89 | 31.61 |
|  | VT18 | 10.70 | 16.12 | 0.11 | 0.06 | 4.74 | 0.30 | 0.63 | 12.60 |
| Average |  | 31.71 | 15.94 | 0.06 | 0.20 | 5.18 | 0.14 | 1.52 | 6.79 |

Table S8. Percentage of concentrations of metals in exchangeable (F1) and bound to carbonates (F2) fractions over the pseudototal fraction in the sediments of Vetas river catchment, Colombia according to the sequential extraction procedure proposed by Tessier et al. (1979).

|  |  | Fe | Mn | Cd | Cr | Cu | Ni | Pb | Zn |
| --- | --- | --- | --- | --- | --- | --- | --- | --- | --- |
| Exchangeable  (%) | VT01 | 0.01 | 1.57 | 13.3 | 0.0 | 0.2 | 0.9 | 0.2 | 1.4 |
|  | VT02 | 0.02 | 3.90 | 8.2 | 0.0 | 0.8 | 1.6 | 1.0 | 5.6 |
|  | VT03 | 0.00 | 1.43 | 4.3 | 0.0 | 0.1 | 0.9 | 0.1 | 1.0 |
|  | VT04 | 0.02 | 25.51 | 19.2 | 0.0 | 0.3 | 1.2 | 0.3 | 3.0 |
|  | VT05 | 0.33 | 18.58 | 1.0 | 0.9 | 1.2 | 8.1 | 6.6 | 9.3 |
|  | VT06 | 0.15 | 3.23 | 1.1 | 0.0 | 0.3 | 3.0 | 4.2 | 1.4 |
|  | VT07 | 0.00 | 1.65 | 7.2 | 0.0 | 0.3 | 0.3 | 1.6 | 2.1 |
|  | VT08 | 0.00 | 2.38 | 3.8 | 0.0 | 0.3 | 0.5 | 0.0 | 3.4 |
|  | VT09 |  |  |  |  |  |  |  |  |
|  | VT10 | 0.01 | 2.90 | 2.5 | 0.0 | 0.3 | 0.9 | 0.3 | 2.3 |
|  | VT11 | 0.01 | 8.16 | 5.4 | 0.0 | 0.6 | 0.9 | 0.1 | 3.8 |
|  | VT12 | 0.01 | 0.28 | 16.5 | 0.1 | 1.3 | 0.8 | 0.9 | 0.6 |
|  | VT13 |  |  |  |  |  |  |  |  |
|  | VT14 | 0.22 | 9.29 | 4.6 | 0.1 | 9.0 | 5.8 | 1.4 | 5.3 |
|  | VT15 | 0.01 | 12.93 | 5.3 | 0.0 | 0.5 | 2.1 | 0.4 | 10.5 |
|  | VT16 | 0.00 | 24.49 | 11.9 | 0.0 | 1.6 | 3.9 | 0.1 | 12.1 |
|  | VT17 | 0.00 | 4.27 | 5.3 | 0.0 | 0.1 | 0.5 | 0.0 | 0.4 |
|  | VT18 | 0.00 | 1.15 | 14.5 | 0.0 | 0.2 | 0.7 | 0.0 | 1.2 |
| Average |  | 0.05 | 7.61 | 7.76 | 0.08 | 1.07 | 2.02 | 1.07 | 3.97 |
| Bound to Carbonates (%) | VT01 | 0.03 | 0.82 | 1.65 | 0.28 | 1.08 | 0.18 | 0.96 | 0.46 |
|  | VT02 | 0.09 | 0.71 | 0.05 | 0.20 | 1.15 | 0.00 | 0.73 | 0.24 |
|  | VT03 | 0.05 | 1.30 | 0.20 | 0.04 | 1.13 | 0.43 | 0.48 | 0.78 |
|  | VT04 | 0.36 | 3.98 | 1.35 | 0.18 | 1.02 | 0.07 | 0.21 | 0.18 |
|  | VT05 | 0.05 | 0.57 | 0.00 | 0.40 | 0.29 | 0.15 | 0.40 | 0.10 |
|  | VT06 | 0.16 | 0.25 | 0.00 | 0.39 | 0.34 | 0.20 | 4.67 | 0.17 |
|  | VT07 | 0.01 | 2.72 | 0.22 | 0.00 | 0.89 | 0.15 | 18.67 | 1.82 |
|  | VT08 | 0.04 | 2.84 | -0.04 | 0.01 | 1.08 | 0.82 | 0.54 | 1.92 |
|  | VT09 |  |  |  |  |  |  |  |  |
|  | VT10 | 0.13 | 0.93 | 0.56 | 0.11 | 2.48 | 1.26 | 3.81 | 2.93 |
|  | VT11 | 0.18 | 8.12 | 3.26 | 0.29 | 3.12 | 1.78 | 0.50 | 7.16 |
|  | VT12 | 0.02 | 1.52 | 7.60 | 0.15 | 1.46 | 0.28 | 2.05 | 0.72 |
|  | VT13 |  |  |  |  |  |  |  |  |
|  | VT14 | 0.13 | 0.37 | 0.00 | 0.06 | 0.77 | 0.34 | 0.34 | 0.08 |
|  | VT15 | 0.03 | 3.01 | 0.00 | 0.18 | 1.34 | 0.85 | 0.78 | 3.81 |
|  | VT16 | 0.09 | 1.85 | 0.77 | 0.14 | 3.00 | 1.02 | 0.24 | 3.15 |
|  | VT17 | 0.11 | 10.14 | 3.74 | 0.33 | 7.91 | 2.45 | 0.40 | 6.54 |
|  | VT18 | 0.03 | 1.63 | 4.11 | 0.05 | 3.26 | 0.94 | 0.83 | 3.41 |
| Average |  | 0.09 | 2.55 | 1.47 | 0.18 | 1.89 | 0.68 | 2.22 | 2.09 |

Table S9. Sum of the concentrations of metals in exchangeable (F1) and bound to carbonates (F2) fractions and percentage of the same sum over the pseudototal fraction in the sediments of Vetas river catchment, Colombia according to the sequential extraction procedure proposed by Tessier et al. (1979).

|  |  | Fe | Mn | Cd | Cr | Cu | Ni | Pb | Zn |
| --- | --- | --- | --- | --- | --- | --- | --- | --- | --- |
| Exchangeable + Bound to carboantes  (mg/L) | VT01 | 9.20 | 24.62 | 0.10 | 0.26 | 0.67 | 0.23 | 0.53 | 1.45 |
|  | VT02 | 18.10 | 15.11 | 0.06 | 0.15 | 0.26 | 0.21 | 0.29 | 2.67 |
|  | VT03 | 20.52 | 38.79 | 0.03 | 0.04 | 1.11 | 0.31 | 0.23 | 3.98 |
|  | VT04 | 95.29 | 139.01 | 0.14 | 0.16 | 0.18 | 0.25 | 0.14 | 1.62 |
|  | VT05 | 160.00 | 98.15 | 0.11 | 1.32 | 6.56 | 7.50 | 23.29 | 40.42 |
|  | VT06 | 118.19 | 64.24 | 0.10 | 0.47 | 1.96 | 3.16 | 26.56 | 15.14 |
|  | VT07 | 5.70 | 55.44 | 0.05 | 0.00 | 0.50 | 0.15 | 0.18 | 4.02 |
|  | VT08 | 15.07 | 70.53 | 0.03 | 0.01 | 0.38 | 0.17 | 0.10 | 5.23 |
|  | VT09 |  |  |  |  |  |  |  |  |
|  | VT10 | 46.03 | 58.29 | 0.12 | 0.15 | 3.16 | 0.75 | 4.09 | 21.11 |
|  | VT11 | 84.58 | 29.75 | 1.02 | 0.45 | 17.46 | 0.28 | 0.99 | 42.42 |
|  | VT12 | 6.68 | 15.07 | 0.16 | 0.26 | 0.39 | 0.19 | 0.45 | 0.80 |
|  | VT13 |  |  |  |  |  |  |  |  |
|  | VT14 | 115.53 | 9.10 | 0.03 | 0.41 | 13.77 | 0.93 | 1.18 | 2.95 |
|  | VT15 | 17.78 | 16.03 | 0.04 | 0.23 | 5.97 | 0.34 | 0.95 | 37.68 |
|  | VT16 | 33.46 | 47.72 | 0.27 | 0.18 | 13.70 | 0.58 | 0.81 | 30.77 |
|  | VT17 | 42.58 | 88.00 | 0.69 | 0.41 | 41.81 | 0.46 | 0.89 | 33.59 |
|  | VT18 | 11.59 | 27.49 | 0.50 | 0.06 | 4.98 | 0.53 | 0.66 | 17.18 |
| Average |  | 50.02 | 49.83 | 0.22 | 0.28 | 7.05 | 1.00 | 3.83 | 16.31 |
| Exchangeable + Bound to carboantes (%) | VT01 | 0.03 | 2.38 | 15.0 | 0.33 | 1.30 | 1.09 | 1.14 | 1.85 |
|  | VT02 | 0.11 | 4.61 | 8.2 | 0.24 | 1.91 | 1.59 | 1.73 | 5.80 |
|  | VT03 | 0.05 | 2.73 | 4.5 | 0.04 | 1.24 | 1.35 | 0.54 | 1.82 |
|  | VT04 | 0.38 | 29.50 | 20.6 | 0.19 | 1.37 | 1.31 | 0.53 | 3.22 |
|  | VT05 | 0.38 | 19.15 | 1.0 | 1.25 | 1.53 | 8.21 | 7.02 | 9.44 |
|  | VT06 | 0.32 | 3.49 | 1.1 | 0.39 | 0.65 | 3.24 | 8.86 | 1.61 |
|  | VT07 | 0.02 | 4.37 | 7.4 | 0.00 | 1.14 | 0.40 | 20.23 | 3.88 |
|  | VT08 | 0.04 | 5.21 | 3.7 | 0.01 | 1.35 | 1.35 | 0.54 | 5.34 |
|  | VT09 |  |  |  |  |  |  |  |  |
|  | VT10 | 0.14 | 3.83 | 3.1 | 0.11 | 2.76 | 2.12 | 4.13 | 5.28 |
|  | VT11 | 0.19 | 16.27 | 8.7 | 0.30 | 3.77 | 2.73 | 0.57 | 10.94 |
|  | VT12 | 0.02 | 1.80 | 24.1 | 0.30 | 2.76 | 1.10 | 2.94 | 1.35 |
|  | VT13 |  |  |  |  |  |  |  |  |
|  | VT14 | 0.35 | 9.66 | 4.6 | 0.18 | 9.76 | 6.18 | 1.70 | 5.39 |
|  | VT15 | 0.04 | 15.93 | 5.3 | 0.18 | 1.84 | 3.00 | 1.13 | 14.30 |
|  | VT16 | 0.09 | 26.34 | 12.6 | 0.14 | 4.57 | 4.92 | 0.35 | 15.26 |
|  | VT17 | 0.11 | 14.41 | 9.0 | 0.33 | 7.99 | 2.99 | 0.40 | 6.95 |
|  | VT18 | 0.03 | 2.78 | 18.6 | 0.05 | 3.43 | 1.65 | 0.87 | 4.65 |
| Average |  | 0.14 | 10.15 | 9.22 | 0.25 | 2.96 | 2.70 | 3.29 | 6.07 |

Table S10. Biovailable fraction toxicity factors (BTfs) and biovailable fraction toxicity index (BTI) in the sediments of the Vetas River, colombia.

|  |  | Cd | Cr | Cu | Ni | Pb | Zn |
| --- | --- | --- | --- | --- | --- | --- | --- |
| Biovailable fraction toxicity factors (BTfs) | VT01 | 0.103 | 0.006 | 0.021 | 0.010 | 0.015 | 0.012 |
|  | VT02 | 0.056 | 0.003 | 0.008 | 0.009 | 0.008 | 0.022 |
|  | VT03 | 0.031 | 0.001 | 0.035 | 0.013 | 0.006 | 0.033 |
|  | VT04 | 0.141 | 0.004 | 0.006 | 0.011 | 0.004 | 0.013 |
|  | VT05 | 0.114 | 0.030 | 0.208 | 0.331 | 0.650 | 0.334 |
|  | VT06 | 0.103 | 0.011 | 0.062 | 0.139 | 0.742 | 0.125 |
|  | VT07 | 0.051 | 0.000 | 0.016 | 0.007 | 0.005 | 0.033 |
|  | VT08 | 0.026 | 0.000 | 0.012 | 0.007 | 0.003 | 0.043 |
|  | VT09 |  |  |  |  |  |  |
|  | VT10 | 0.121 | 0.003 | 0.100 | 0.033 | 0.114 | 0.174 |
|  | VT11 | 1.007 | 0.010 | 0.553 | 0.013 | 0.028 | 0.351 |
|  | VT12 | 0.166 | 0.006 | 0.012 | 0.009 | 0.012 | 0.007 |
|  | VT13 |  |  |  |  |  |  |
|  | VT14 | 0.031 | 0.009 | 0.436 | 0.041 | 0.033 | 0.024 |
|  | VT15 | 0.036 | 0.005 | 0.189 | 0.015 | 0.026 | 0.311 |
|  | VT16 | 0.272 | 0.004 | 0.433 | 0.025 | 0.023 | 0.254 |
|  | VT17 | 0.702 | 0.009 | 1.087 | 0.020 | 0.025 | 0.278 |
|  | VT18 | 0.508 | 0.001 | 0.158 | 0.023 | 0.018 | 0.142 |
| Average |  | 0.217 | 0.007 | 0.208 | 0.044 | 0.107 | 0.135 |
| Biovailable fraction toxicity index (BTI) | VT01 | VT02 | VT03 | VT04 | VT05 | VT06 | VT07 |
|  | 0.017 | 0.012 | 0.012 | 0.011 | 0.193 | 0.098 | 0.000 |
|  | VT08 | VT09 | VT10 | VT11 | VT12 | VT13 | VT14 |
|  | 0.006 |  | 0.055 | 0.094 | 0.014 |  | 0.040 |
|  | VT15 | VT16 | VT17 | VT18 |  |  |  |
|  | 0.040 | 0.064 | 0.100 | 0.044 |  |  |  |


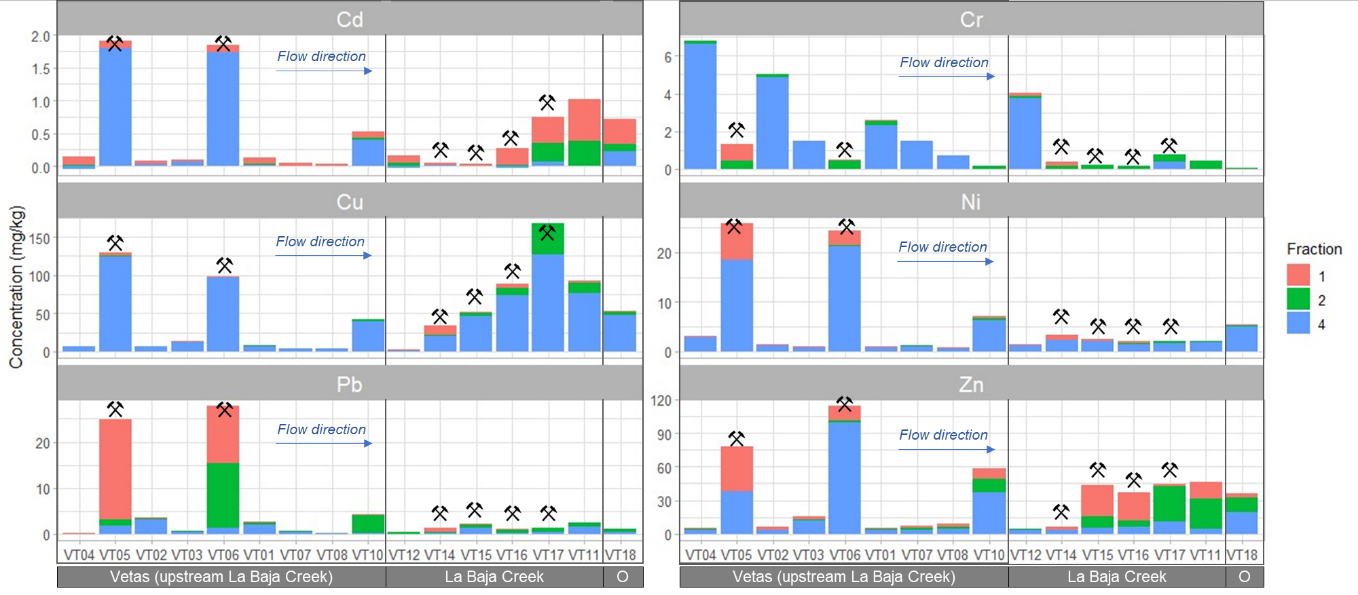


Figure S7. Concentration of Cd, Cr, Cu, Ni, Pb and Zn in fractions 1 (red), 2 (green) and 4 (blue) in sediment samples of Vetas River basin, Colombia according to the sequential extraction procedure proposed by Tessier et al. (1979).
